# Morpholine Nitrogen and Oxygen Atoms as Halogen Bond Acceptors in Different Environments: Cocrystals of Amino‑, Imino- or Amide-Linked Bis(morpholine) Derivatives with Perfluorinated Iodohydrocarbons

**DOI:** 10.1021/acs.cgd.6c00316

**Published:** 2026-06-18

**Authors:** Kristina Smokrović, Nikola Bedeković, Vladimir Stilinović

**Affiliations:** † Jožef Stefan Institute, Jamova cesta 39, 1000 Ljubljana, Slovenia; ‡ Department of Chemistry, Faculty of Science, 117036 University of Zagreb, Horvatovac 102a, 10000 Zagreb, Croatia

## Abstract

The propensity of the morpholine oxygen as a halogen bond acceptor in the presence of potentially competing halogen bond acceptor groups was studied by preparing a series of three bis­(morpholine) derivatives in which the morpholine rings are interconnected via diamine, diimine, and diamide linkers and cocrystallizing them with an excess of 1,2-diiodotetrafluorobenzene, 1,3-diiodotetrafluorobenzene, 1,4-diiodotetrafluorobenzene, 1,3,5-triiodotrfluorobenzene, and 1,4-diiodooctafluorobutane as halogen bond donors. This yielded a total of 11 cocrystals. Both the diamide and the diimine yielded four cocrystals (with all the aromatic donors), with morpholine oxygen atoms participating in halogen bonding in only two diamide cocrystals and in all four diimines, reflecting the more negative electrostatic potential of the amide nitrogen in comparison to the imide. The diamide acceptor, however, formed only three cocrystals, all three featuring halogen bonds with morpholine oxygen as well as amide oxygen atoms, despite amide oxygen being the most negative site. This can generally be attributed to competing interactions, such as hydrogen bonding of a water molecule to the amide oxygen as well as general packing effects.

## Graphical abstract

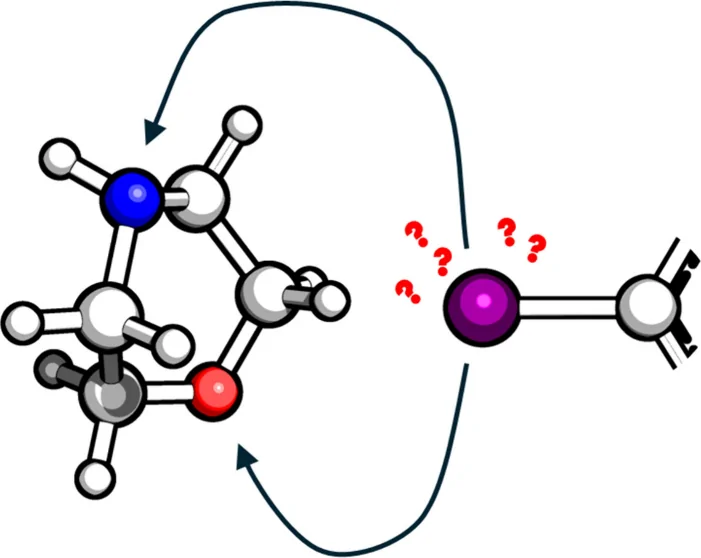

## Introduction

Over the recent decades, the halogen bond has become one of the most important intermolecular interactions (second only to the hydrogen bond) in supramolecular chemistry and crystal engineering, particularly.^1^ Among Lewis bases employed as halogen bond acceptors, by far the most common have been nitrogen atoms within aromatic cyclic molecules, particularly pyridine derivatives,^2^ which have proven to be a valuable asset for comparison and evaluation of halogen bond donors.^3,4^ In recent years, they have been followed by a variety of other, mostly nitrogen- or oxygen-atom-containing species, such as methoxy,^5–8^ nitro,^9–12^ hydroxyl,^13^ and nitrile^7,14^ functional groups and oxygen atoms in *N*-oxides.^15–18^ Some studies have also showcased the promising halogen bond acceptor potential of nitrogen atoms in piperazine^16–19^ as well as the carbonyl oxygen atom.^20^

One case where both nitrogen and oxygen atoms are present in the molecule and both act as halogen bond acceptors is morpholine, where even though the nitrogen site is more nucleophilic, both heteroatoms act as halogen bond acceptors with donors such as iodoalkynes^21,22^ and perfluorinated iodobenzenes.^23,24^ However, as the nitrogen atom is the more nucleophilic site, it can coordinate metal cations, forming complexes in which only the oxygen atom remains free for halogen bonding.^25^ The nucleophilicity of the morpholine nitrogen is also a reactive site for (electrophilic) substitution of the hydrogen atom with other atoms and functional groups. This allows for very simple derivatization of morpholine to various imines,^26–31^ amines,^32–35^ carbonyl compounds, etc.^36–41^ Such derivatization will unavoidably modify the ability of the nitrogen atom to participate in the intermolecular interactions. However, it should leave the oxygen atom on the periphery as a potential halogen bond acceptor. Recently, our group demonstrated that the hydrogen-donating ability of the morpholine oxygen can be preserved from a very simple morpholine derivative (*N*-aminomorpholine) through a series of derivatizations.^17,26,27^

In the present study, we decided to explore more systematically how various modes of morpholine derivatization (Scheme 1) affect the probability that the morpholine oxygen will act as a halogen bond acceptor in cocrystals with a series of halogen bond donors. For this purpose, we prepared three bis­(morpholine) derivatives: a tertiary amine (**1**), an imine (**2**), and an amide (**3**). These three different acceptors include spacers of variable rigidity and length: an alkyl chain of two methylene groups (**1**), a four-atom chain containing two imine groups (**2**), and a three-atom chain containing two carboxyl groups and one methylene group (**3**). The amine acceptor **1** contains two possible acceptor atoms: the amine nitrogen atom and the morpholinyl oxygen atom. The imine acceptor **2** and amide receptor **3** both contain three possible acceptor atoms: morpholinyl oxygen, imine nitrogen, and amine nitrogen atoms in **2** and morpholinyl oxygen, amide oxygen, and amide nitrogen atoms in **3**. These three acceptors were crystallized with 1,2,3,4-tetrafluoro-5,6-diiodobenzene (**1,2-tfib**), 1,2,3,5-tetrafluoro-4,6-diiodobenzene (**1,3-tfib**), 1,2,3,5-tetrafluoro-3,6-diiodobenzene (**1,4-tfib**), 1,3,5-trifluoro-2,4,6-triiodobenzene (**1,3,5-tfib**), and 1,1,2,2,3,3,4,4-octafluoro-1,4-diiodobutane (**1,4-ofib**) to observe the behavior of the selected acceptors in the presence of halogen bond donors with different geometries (**1,2-tfib**, **1,3-tfib**, **1,4-tfib**), topicities (with tritopic **1,3,5-tfib**), and flexibilities (with flexible aliphatic **1,4-ofib**).

**Scheme 1:**
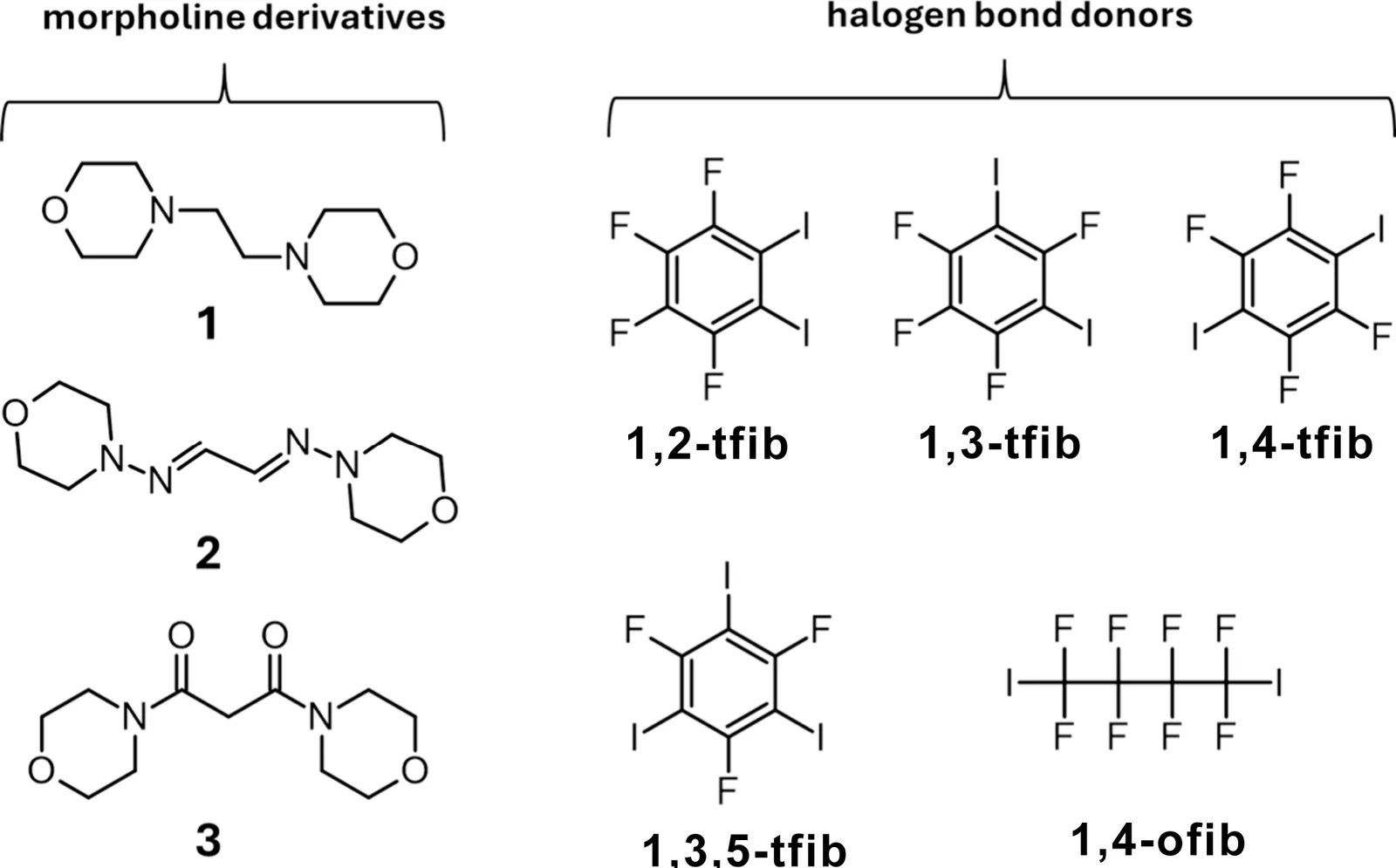
Structural Diagrams of the Studied Morpholine Derivatives **1**–**3** and the Selected Halogen Bond Donors

## Results and Discussion

In order to evaluate bis­(morpholine) derivatives **1**–**3** as potential halogen bond (XB) acceptors, we computed the molecular electrostatic potentials (ESPs) on optimized geometries of the three molecules *in vacuo* (Figure 1). As a result of the optimization, local geometric minima were obtained for molecules **1** and **2** in both bent conformations (**1**_bent_/**2**_bent_; Figure 1a,c) and extended conformations (**1**_ex_/**2**_ex_; Figure 1b,d) as well as for molecule **3** in a bent conformation (Figure 1e). The most negative ESP values in both conformations of **1** and **2** are found at the morpholine oxygen atoms, followed by the morpholine nitrogen atoms in molecule **1** and the imine nitrogen atoms in molecule **2**. In **1**, the ESP values at the acceptor sites are more negative in the bent conformation compared to the extended conformation (ΔESP­(O_morph_) = 7.5 kJ mol^–1^ e^–1^; ΔESP­(N_morph_) = 12.2 kJ mol^–1^ e^–1^). In contrast, molecule **2** shows mostly equal ESP values on acceptor sites in both conformations. The only difference is the value on the oxygen atom in the twisted morpholine branch of the **2**_bent_ molecule, which is ca. 2 kJ mol^–1^ more negative than that in **2**_ex_. Despite the more positive ESP values at the acceptor sites, only extended conformations are present in the crystal structures of the cocrystals with **1** and **2**. Those conformations are also more stable in the gas phase compared to the bent conformations, with energy differences of *E*(**1**_ex_) – *E*(**1**_bent_) = −8.8 kJ mol^–1^ and *E*(**2**_ex_) – *E*(**2**_bent_) = −11.6 kJ mol^–1^. In molecule **3**, the most negative site corresponds to the amide oxygen (−137 and −143 kJ mol^–1^ e^–1^), followed by morpholine oxygen atoms (−109 and −115 kJ mol^–1^ e^–1^), while morpholine nitrogen atoms do not correspond to negative sites, and are therefore not expected to act as halogen bond acceptors.

**Figure 1:**
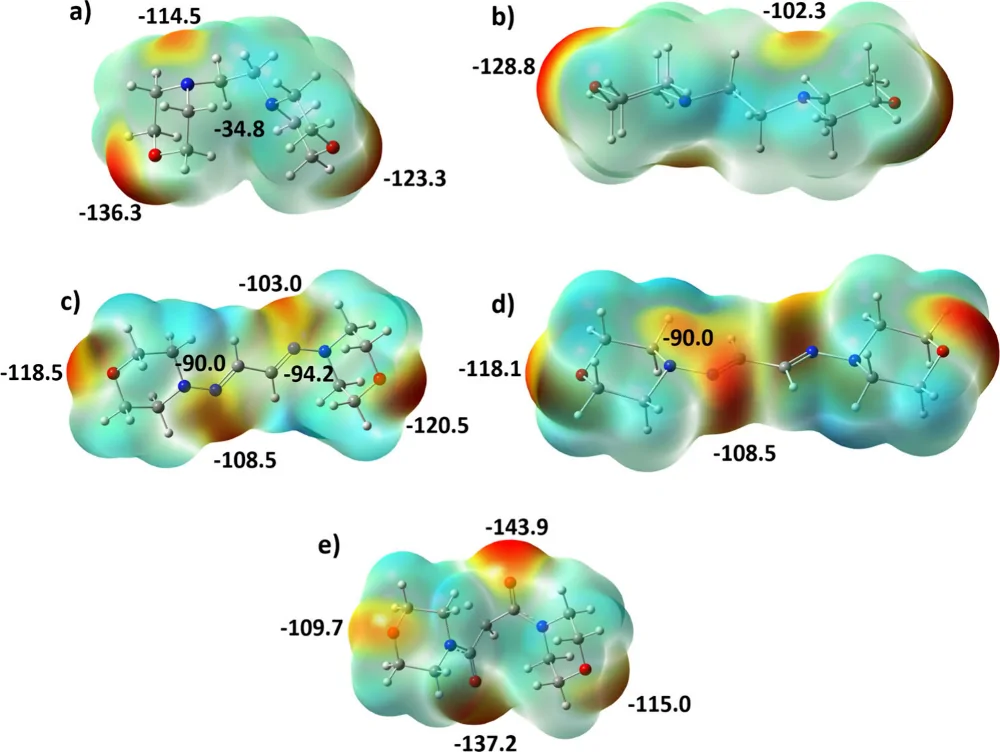
Electrostatic potentials (kJ mol^–1^ e^–1^) mapped on 0.001 au electron density isosurfaces for the optimized molecular geometries of (a) **1**_bent_, (b) **1**_ex_, (c) **2**_bent_, (d) **2**_ex_, and (e) **3**.

Out of 15 possible donor–acceptor combinations, 11 cocrystals were obtained: **1**·(**1,2-tfib**)_2_, **1**·**1,3-tfib**, **1**_2_·**1,4-tfib**, **1**·(**1,3,5-tfib**)_4_, **2**_2_·**1,4-tfib**·2H_2_O, **2**·**1,3,5-tfib**, **2**_2_·**1,4-ofib**, **3**·(**1,2-tfib**)_2_, **3**·(**1,3-tfib**)_2_, **3**·(**1,4-tfib**)_2_, and **3**·(**1,3,5-tfib**)_2_ (Table 1). Also, two forms of the amide **2** were obtained, an anhydrous form and the hydrate **2**·H_2_O, as byproducts during the cocrystallization experiments.

**Table 1:** Overview of Obtained Cocrystals and Geometric Parameters (Length (*d*), Relative Shortening (RS), and Angle (α)) of the Halogen Bonds Observed in Their Structures

compound	acceptor atom (A)	*d*(A···I) (Å)	RS (%)^a^	α(A···I–C) (deg)
**1**·(**1,2-tfib**)_2_	N_amine_	2.979(4)	15.61	177.11(16)
	O_morpholine_	2.951(4)	15.69	178.92(14)
**1**·**1,3-tfib**	N_amine_	2.912(4)	17.51	173.5(1)
**1**_2_·**1,4-tfib**	N_amine_	2.955(2)	16.29	170.73(8)
**1**·(**1,3,5-tfib**)_4_	N_amine_	2.892(7)	18.07	172.5(3)
	O_morpholine_	2.921(7)	16.54	177.1(3)
**2**·(**1,2-tfib**)_2_	N_imine_	3.101(5)	12.15	177.1(2)
	O_morpholine_	2.804(6)	19.89	172.9(2)
**2**·(**1,3-tfib**)_2_	N_imine_	3.122(14)	11.56	165.8(5)
	O_morpholine_	2.939(14)	16.03	176.5(5)
**2**·(**1,4-tfib**)_2_	N_imine_	3.213(3)	8.98	164.6(1)
	O_morpholine_	2.968(4)	15.20	170.3(2)
**2**·(**1,3,5-tfib**)_2_	N_morpholine_	3.245(8)	8.07	168.0(3)
	N_imine_	3.303(9)	6.43	165.0(3)
		3.037(8)	13.97	175.3(3)
	O_morpholine_	3.078(8)	12.06	164.9(3)
		2.959(8)	15.46	176.8(3)
	I	3.9354(11)	0.62	163.6(3)
		3.8720(12)	2.22	109.8(3)
**3**_2_·**1,4-tfib**·2H_2_O	O_morpholine_	2.895(3)	17.29	168.5(1)
**3**·**1,3,5-tfib**	O_morpholine_	2.902(5)	17.09	169.8(2)
	O_carbonyl_	2.982(4)	14.80	170.2(2)
	I	3.8146(7)	3.67	164.2(1)
**3**_2_·**1,4-ofib**	O_morpholine_	2.898(5)	17.20	178.5(2)

a RS = {1 – *d*(I···A)/[*r*_vdW_(I) + *r*_vdW_(A)]} × 100%.

In all the obtained cocrystals, the amine acceptor **1** adopted the extended conformation, with the central spacer in the *anti*-conformation (the torsion angle between morpholine substituents is 180°). This allowed the XB donor to approach both nitrogen and oxygen morpholine atoms without steric hindrance. Acceptor **1** formed cocrystals with all the donors except for **1,4-ofib**, with various stoichiometries and supramolecular structures (Figure 2). In all four studied cocrystals, the amine nitrogen atoms of **1** were found to act as halogen bond acceptors, while the morpholine oxygen atoms were involved in halogen bonding in only two (**1**·(**1,2-tfib**)_2_ and **1**·(**1,3,5-tfib**)_4_). This is somewhat unexpected from the viewpoint of the molecular electrostatic potential, as the oxygen atoms correspond to more negative ESP values (Figure 1). This could indicate the preference of iodine (as a soft acid) for nitrogen (a softer base than oxygen). However, this could also be the result of overall crystal packing efficiency. Indeed, the fact that in all four cocrystals the molecules of **1** adopt the extended conformation (which is not the most stable in the gas phase but is capable of more efficient packing) as well as the somewhat unexpected stoichiometries in the crystal structures of two cocrystals (**1**_2_·**1,4-tfib** and **1**·(**1,3,5-tfib**)_4_; see discussion below) in particularly seem to give credence to the latter explanation.

**Figure 2:**
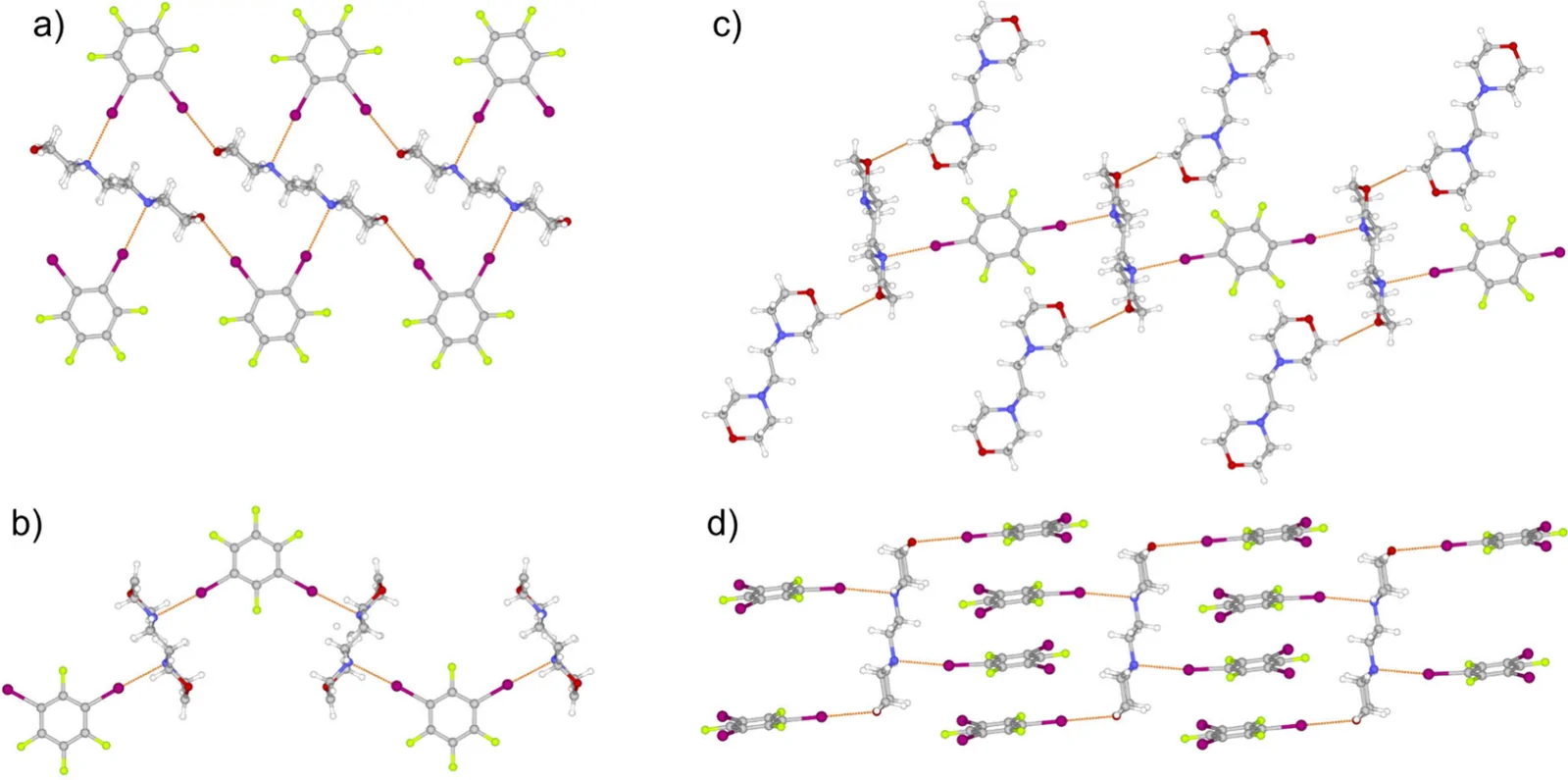
Supramolecular motifs in the crystal structures of cocrystals of diamine acceptor **1** with halogen bond donors: (a) halogen-bonded double chains in **1**·(**1,2-tfib**)_2_; (b) halogen-bonded single chains in **1**·**1,3-tfib**; (c) halogen-bonded single chains in **1**_2_·**1,4-tfib** (with the C–H···O-bonded extra molecules of **1**·**1,4-tfib**); (d) close packing of discrete **1**·(**1,3,5-tfib**)_4_ supramolecular complexes.

With **1,2-tfib**, a cocrystal of **1**·(**1,2-tfib**)_2_ stoichiometry was obtained in which one molecule of **1** participates in four halogen bonds with four separate **1,2-tfib** molecules, forming infinite double chains (Figure 2a). Each **1,2-tfib** molecule participates in a halogen bond with an amine nitrogen atom and a morpholinyl oxygen atom of **1**. The crystal structure of **1**·**1,3-tfib** comprises a simple chain motif (Figure 2b), where each acceptor molecule is connected with two halogen bond donor molecules via the amine nitrogen atoms. A similar halogen-bonded chain motif was found to be present in the cocrystal **1**_2_·**1,4-tfib**. Here, however, for each halogen-bonded pair of **1** and the donor, there is one additional molecule of **1** which does not participate in any halogen bond and interacts with the halogen-bonded molecule of **1** only via C–H···O hydrogen bonds (Figure 2c), which interconnect the chains into a 3D structure. It is important to note that, as in the other cocrystal syntheses, here the reaction mixture was such that the donor was present in a large excess—more than sufficient to satiate all available acceptor atoms. The fact that from such a mixture the crystal obtained comprises acceptor molecules not involved in halogen bonding at all is quite indicative of particular stabilization of the crystal structure by the presence of the “free” molecules of **1**.

The pattern of supramolecular halogen-bonded chains in cocrystals of **1** was broken by the cocrystal with **1,3,5-tfib**, which formed a discrete supramolecular complex **1**·(**1,3,5-tfib**)_4_. Each molecule of the acceptor is coordinated with four halogen bonds and four **1,3,5-tfib** molecules. This created a double-pronged supramolecular complex with the **1,3,5-tfib** molecules fitting into the crevices of the neighboring complex and connected only via I···π contacts. The fact that each **1,3,5-tfib** molecule—a potentially tritopic donor—formed only one halogen bond with the acceptor molecule is in accord with earlier observation that **1,3,5-tfib** generally forms only one or two halogen bonds with weaker bases.^3^ However, statistically the most likely topicity of **1,3,5-tfib** in cocrystals with polytopic acceptors is two (as indeed is the case in cocrystals of **2** and **3**). The fact that here it acts only as a monotopic halogen bond donor is likely due to the fact that the self-complementary shape of the **1**·(**1,3,5-tfib**)_4_ supramolecular complex (with protruding **1,3,5-tfib** molecules and “empty slots” between them) is well-suited for efficient filling of space.

The diimine acceptor **2**, like **1**, was found to form cocrystals with all the halogen bond donors, having similar structural motifs. This is to be expected since acceptor **2** also adopts a fully extended conformation, with the torsion angle of the linker chain being 180(6)° and the morpholine rings in chair conformation (except for the structure **2**·**1,3,5-tfib**, where the torsion angle is 173(1)°). However, there is a notable difference in the preferred XB acceptor sites compared to **1**: in all four cocrystals the morpholine oxygen atoms are halogen bond acceptors, alongside with the (imine) nitrogen. Thus, double chains equivalent to those present in **1**·(**1,2-tfib**)_2_ were also observed in two cocrystals of **2**, namely, **2**·(**1,2-tfib**)_2_ and **2**·(**1,3-tfib**)_2_ (Figure 3), only involving different sized supramolecular rings. The cocrystal of **1,2-tfib** forms infinite halogen-bonded chains in which two molecules of **1** are bridged by two donor **1,2-tfib** molecules connected into rings.

**Figure 3:**
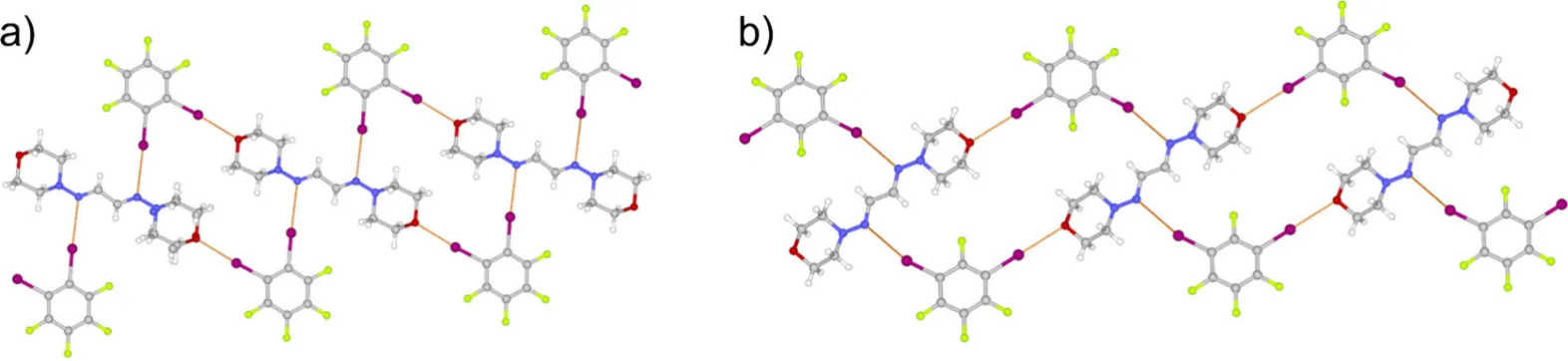
Halogen-bonded double chains in (a) **2**·(**1,2-tfib**)_2_ and (b) **2**·(**1,3-tfib**)_2_.

The crystal structure of **2**·(**1,4-tfib**)_2_, while having the same stoichiometric ratio of donors and acceptors as the cocrystals of **2** with **1,2-tfib** and **1,3-tfib**, has a markedly different structure. Here, each acceptor molecule binds four separate donor molecules. Of the two donor molecules that are independent by symmetry, one forms a pair of I···O halogen bonds with morpholine oxygen atoms of two neighboring acceptor molecules while the other bridges two acceptor molecules via two I···N halogen bonds with the imine nitrogen atoms, forming a 2D supramolecular network (Figure 4a).

**Figure 4:**
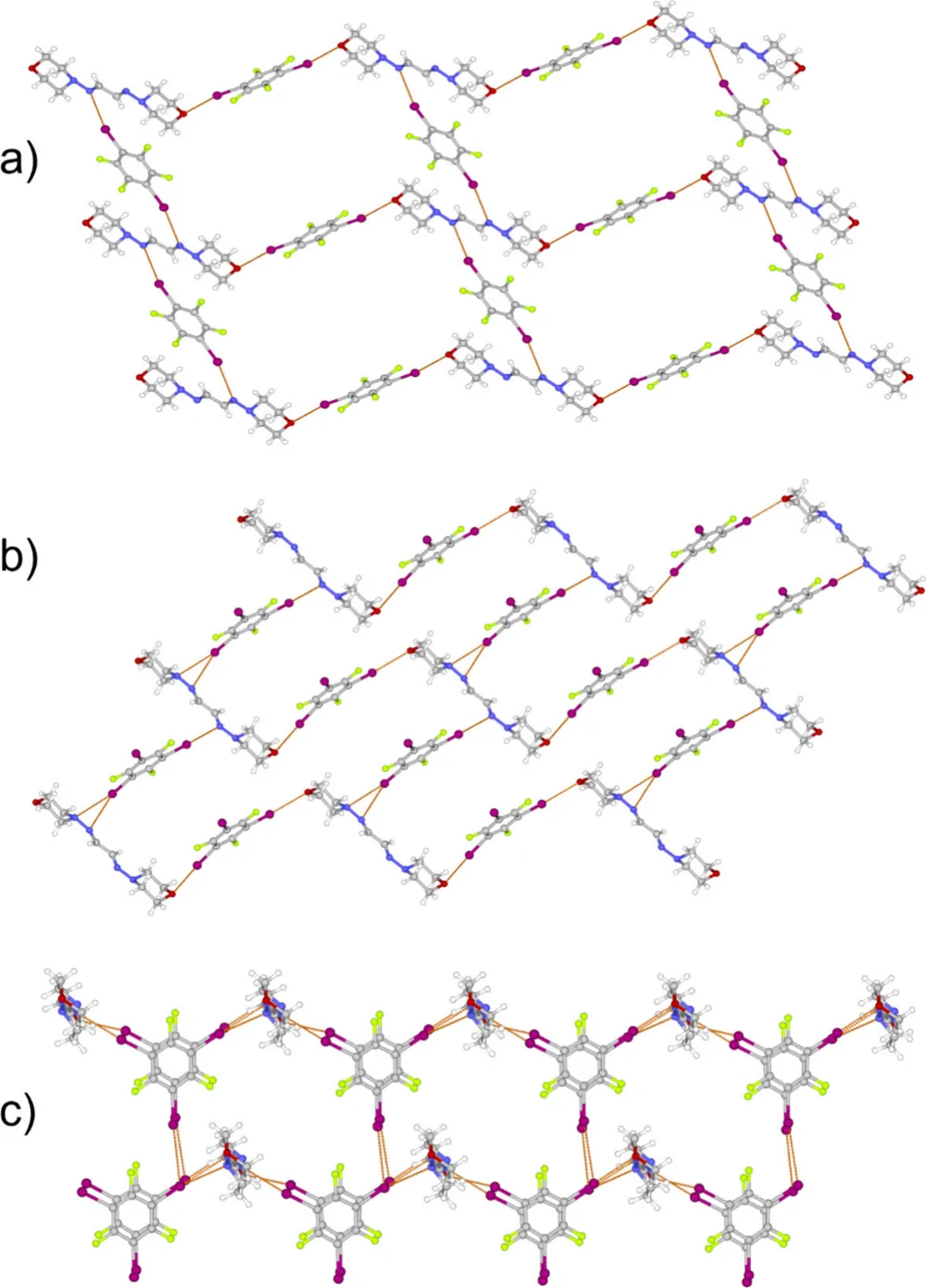
Fragments of halogen-bonded networks in (a) **2**·(**1,4-tfib**)_2_ and (b, c) **2**·(**1,3,5-tfib**)_2_. (a) 2D networks in **2**·(**1,4-tfib**)_2_. (b) Halogen bond ring motifs connected into networks observed in the crystal structures of **2** with **1,4-tfib** and **1,3,5-tfib**. (a) cocrystallization with **1,4-tfib** results in a 2D network. **1,3,5-tfib** yields a 3D network where (b) N···I and O···I XB bonds form a 2D network in the (100) plane, which are then interconnected by (c) I···I bonds perpendicular to the (100) plane.

A similar motif is also achieved in **2**·(**1,3,5-tfib**)_2_. Despite the presence of an extra iodine atom on the donor molecule, **1,3,5-tfib** binds to **2** as a ditopic donor (again with one acceptor molecule binding to two morpholine oxygens and the other to nitrogen atoms; Figure 4b). Here, however, the donor molecules lie approximately perpendicular to the network plane, somewhat changing the geometry of the I···N halogen bonds. While one iodine participates in a classical monocentric I···N halogen bond with an imine nitrogen, the other approaches the imine group perpendicular to the mean plane of the central chain and achieves contact with both the imine and the amine nitrogen, forming a bifurcated halogen bond. The remaining iodine atoms of the donor molecules act as donors in C–I···I halogen bonds with the donor molecules from neighboring layers, interconnecting them into a 3D structure (Figure 4c).

The amide acceptor **3** differs from **1** and **2** in two significant ways: the morpholine oxygen atom is not the most negative binding site (the carbonyl oxygen being more negative), and the molecule is markedly asymmetric, as it cannot adopt a symmetric conformation (due to odd-number carbon chain of the central spacer). Accordingly, **3** was found to form a markedly different series of cocrystals than **1** and **2**. On one hand, we were unable to obtain cocrystals with either **1,2-tfib** or **1,3-tfib**, which formed with the previous two acceptors, while on the other, only **3** afforded a cocrystal with the aliphatic XB donor **1,4-ofib**. Here again, the morpholine oxygen atoms were found in all cocrystals to act as halogen bond acceptor sites. However, unlike in the four cocrystals with **2**, where both morpholine oxygen atoms of the donor molecule were involved in halogen bonding, in all three cocrystals obtained with **3**, only one morpholine oxygen per acceptor molecule is involved in halogen bonding with the donor molecules.

The **3**·**1,3,5-tfib** cocrystal was found to comprise discrete centrosymmetric **3**_2_·(**1,3,5-tfib**)_2_ supramolecular tetramers, with each donor molecule forming a halogen bond with a carbonyl oxygen atom of one molecule of **3** and with a morpholine oxygen atom of the other (Figure 5a). The remaining carbonyl and morpholine oxygen atoms participate only in C–H···O contacts, while the third iodine atom of the **1,3,5-tfib** molecule acts as a donor in a fairly long I···I halogen bond (type II interhalogen contact of 3.8146(6) Å; Figure 5b). Thus, despite the presence of an abundance of halogen bond donors in the crystal structure, only one of the carboxyl oxygen atoms (the most negative site of the molecule of **3**) is involved in halogen bonding.

**Figure 5:**
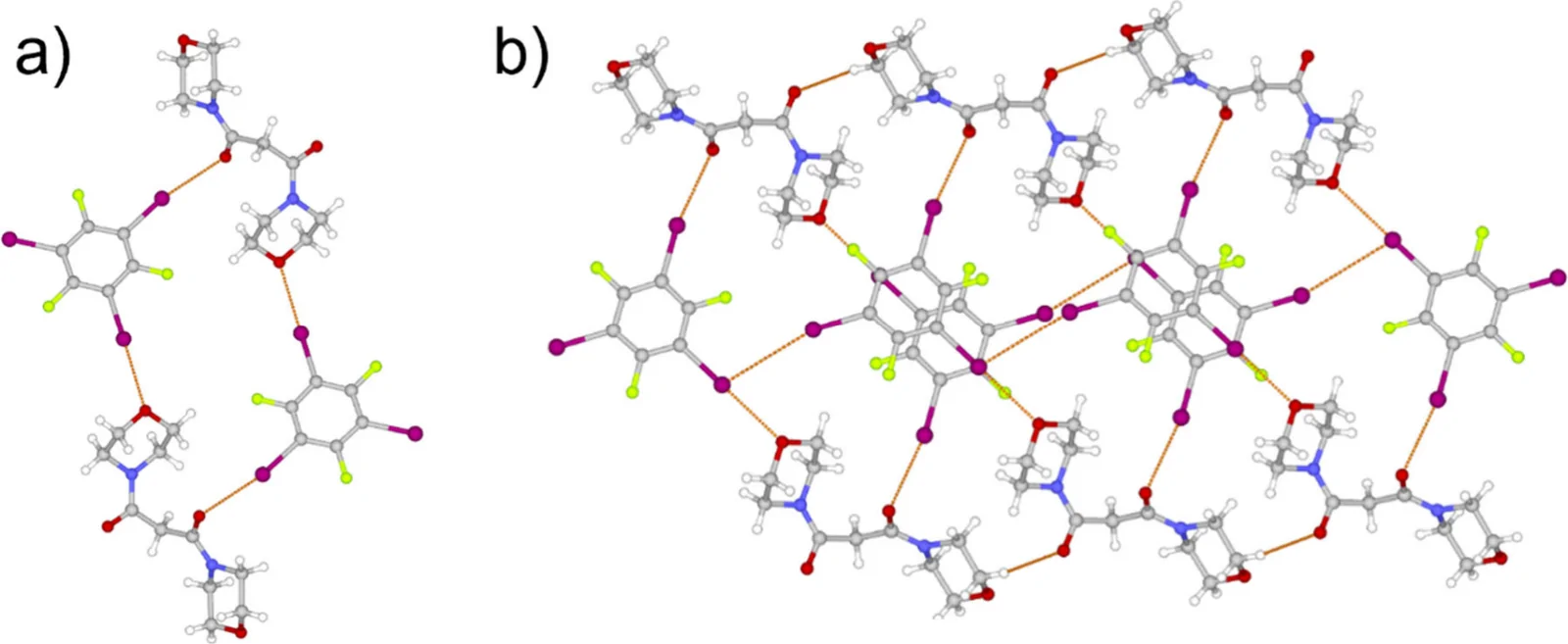
Crystal structure of **3**·**1,3,5-tfib**: (a) halogen-bonded **3**_2_·(**1,3,5-tfib**)_2_ supramolecular tetramers; (b) interconnection of the supramolecular tetramers via I···I interhalogen contacts into chains.

This apparent reluctance of the carbonyl oxygen atoms of **3** to participate in halogen bonding is even more evident in the remaining two structures. The **3**_2_·**1,4-ofib** cocrystal thus comprises discrete centrosymmetric **3**_2_·**1,4-ofib** halogen-bonded trimers where one **1,4-ofib** molecule forms two (identical) I···O halogen bonds with morpholine oxygen atoms of two molecules of **3**, with both the carbonyl oxygen atoms participating solely in C–H···O contacts, interconnecting the trimers into layers perpendicular to [102̅] (Figure 6).

**Figure 6:**
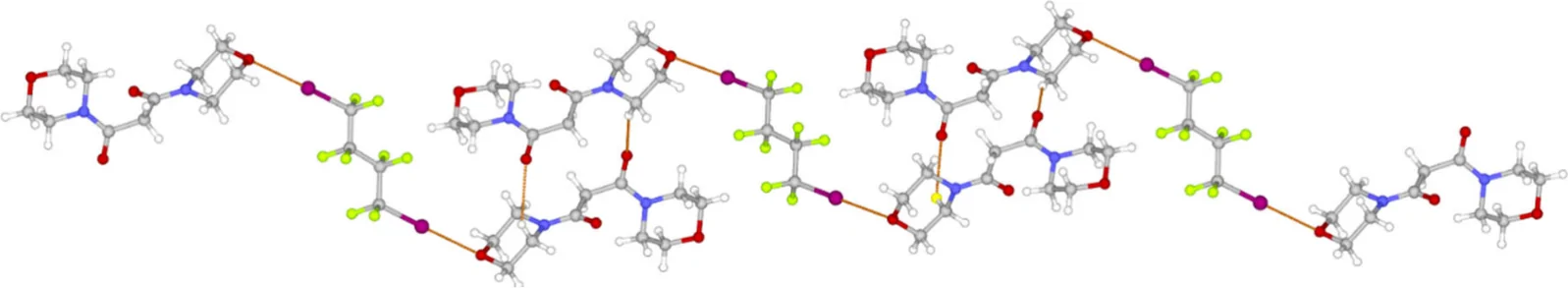
Halogen-bonded discrete units in **3**_2_·**1,4-ofib** connected into chains via C–H···O hydrogen bonds between amide oxygen atoms and the morpholine ring’s methylene hydrogen atoms.

The cocrystal obtained with **1,4-tfib** also features the motif of two molecules of **3** halogen-bonded to a donor molecule solely via one morpholine oxygen atom each (Figure 7). However, this cocrystal is unique among the entire series in that it crystallized as a hydrate, **3**_2_·**1,4-tfib**·2H_2_O. All attempts to crystallize an anhydrous cocrystal failed. On the other hand, a hydrate of the amide (**3**·H_2_O) as well as a solvent-free amide could be obtained. The comparison of the structures of the **3**_2_·**1,4-tfib**·2H_2_O cocrystal and the **3**·H_2_O hydrate provides one of the most beautiful examples of supramolecular synthon preservation in all of the authors’ experience (Figure 7). Both structures comprise almost identical helical hydrogen-bonded chains formed by water molecules acting as hydrogen bond donors to amide oxygen atoms of two molecules of **3**, each amide oxygen forming one hydrogen bond. In **3**·H_2_O these chains (extended along the crystallographic *c* axis) are interconnected by C–H···O contacts between morpholine rings of neighboring chains—one morpholine ring participates in a zigzag chain (*C*_1_^1^(3)) along the *b* axis interconnected by relatively short C–H···O contacts (C4–H4a···O1 of 3.359(3) Å) and the other in two pairs of centrosymmetric C–H···O contacts, each pair forming an *R*_2_^2^(6) motif with somewhat longer C–H···O contacts (C11–H11b···O4 of 3.464(3) Å and C9–H9a···O4 of 3.493(4) Å). In the **3**_2_·**1,4-tfib**·2H_2_O cocrystal, the **1,4-tfib** molecules disrupt only the latter series of long contacts and C–H···O contacts and intercalate between molecules of **3**, forming instead halogen I···O halogen bonds with the morpholine oxygen atoms. The amide oxygen atoms and water molecules form hydrogen-bonded chains that are almost identical to those in **3**·H_2_O. The similar conformations and hydrogen bond parameters (Table 2) allow the preservation of the *C*_1_^1^(3) motif interconnecting them along the *b* axis (with the corresponding C–H···O contact (C11–H11a···O4) of 3.372(5) Å).

**Table 2:** Hydrogen Bond Parameters in the Two Obtained Hydrates **3**·H_2_O and **3**_2_·**1,4-tfib**·2H_2_O

compound	*d*(C=O···OH_2_) (Å)	α(C=O···O···O=C) (deg)	α(O=C···C=O) (deg)
**3**·H_2_O	2.851(3)	102.71(8)	76.8(2)
	2.884(3)		
**3**_2_·**1,4-tfib**·2H_2_O	2.892(4)	106.2(1)	78.1(3)
	2.861(4)		

**Figure 7:**
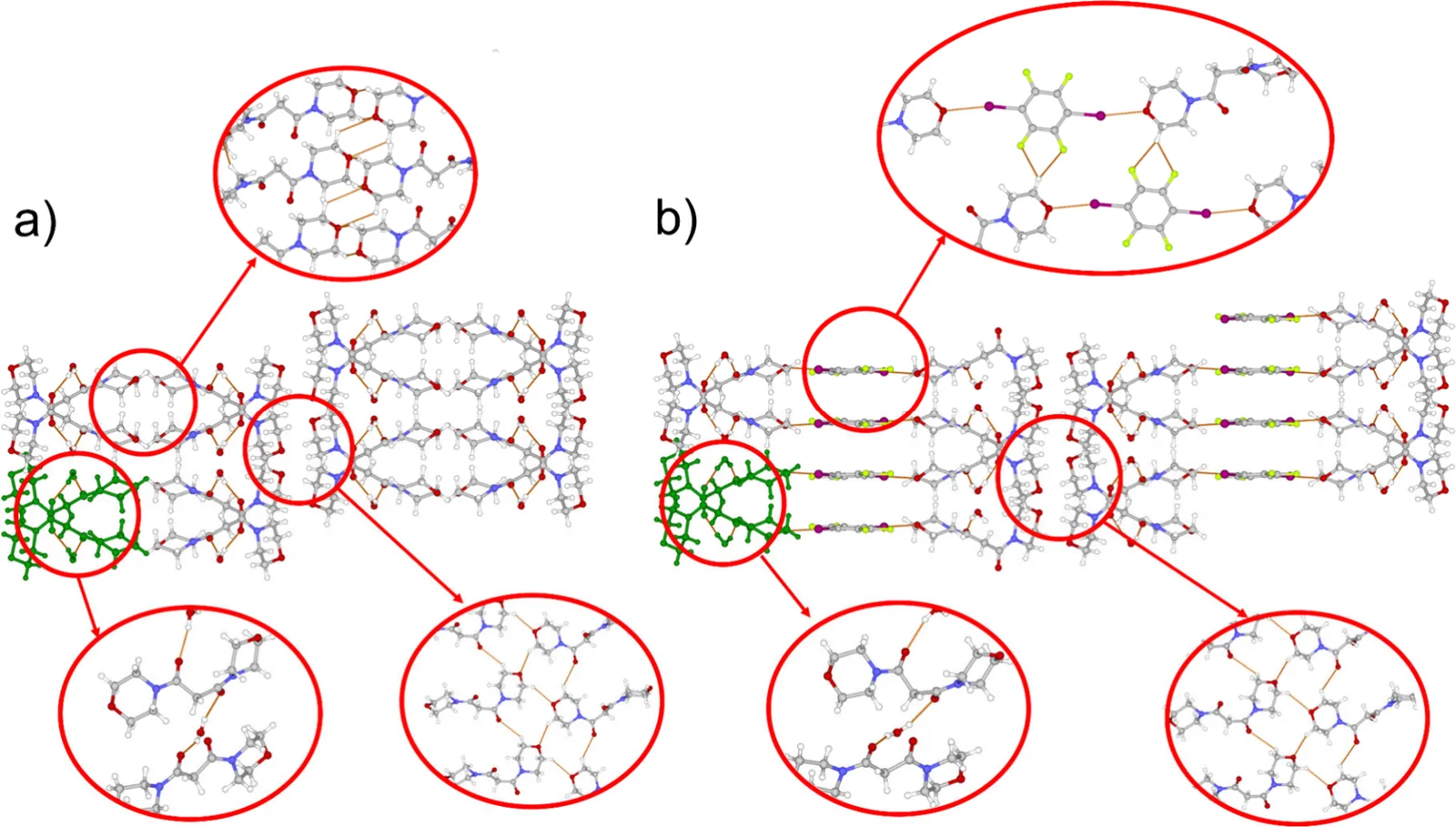
Crystal structures of (a) **3**·H_2_O and (b) **3**_2_·**1,4-tfib**·2H_2_O. Hydrogen bonded chains (bottom left) expanding along the crystallographic *c* axis interconnect into a 3D structure (center—a single chain is highlighted in green) through (a) two C–H···O bonding motifs between morpholine rings in **3**·H_2_O and (b) a C–H···O bonding motif between morpholine rings preserved from the hydrate (bottom right) and a combination of I···O halogen bonds and C–H···F contacts in **3**_2_·**1,4-tfib**·2H_2_O.

The apparent stability of the hydrogen-bonded chains led us to attempt introducing water into the other cocrystals obtained with **3**, but all those attempts were unsuccessful. It seems, therefore, that the formation of the hydrogen-bonded chains is driven not only by the favorability of the hydrogen-bonded chains but also by crystal packing effects. The packing effects are also likely responsible for the apparent preference of the XB donors to interact with the less-negative morpholine oxygen atoms rather than the carbonyl oxygen atoms. Although the carbonyl oxygen would expectedly be a better acceptor site, the sterically more accessible morpholine oxygen interacts with the (large) halogen bond donor molecules largely due to the somewhat awkward shape of the acceptor molecule.The much smaller water molecule then successfully penetrates to the carbonyl oxygen and forms hydrogen bonds with the best (most negative) acceptor sites.

## Conclusion

The morpholine oxygen atom has proven to form halogen bonds fairly reliably with iodine XB donors in the presence of all the tested competing groups, with the O_morph_···I synthon being present in ca. 85% (i.e., 11 out of 13) obtained cocrystals. Interestingly, the only competing group to preclude formation of halogen bonds with the morpholine oxygen was not the amide oxygen in **3**—having the most negative local ESP—but rather the tertiary amine nitrogen in **1**, even though in the molecule of **1** the oxygen atom corresponds to a considerably lower ESP. This possibly reflects the *soft* Lewis acidity of the iodine and its subsequent preference for a *softer* base or is induced by crystal packing effects. On the other hand, the apparent preference of the XB donors to interact with the less negative morpholine oxygen atoms rather than the carbonyl oxygen in **3** can almost certainly be attributed to crystal packing effects. This, however, is not to say that the observed behavior should be regarded as an outlier—the easy access of the donor molecule to the morpholine ring on the molecular periphery (in comparison to the atoms in the linking chain) can always be expected to be an important deciding factor for the formation of intermolecular interactions. Therefore, the introduction of morpholine rings into molecular systems to make them better potential XB acceptors, using various chemical modes available, seems to be a viable strategy for the design of halogen-bonded supramolecular materials, with the caveat that the halogen-bonded architecture which is obtained will be much affected by crystal packing effects.

## Experimental Section

### Synthesis

All halogen bond donors were purchased from Manchester Organics Company; *N*-aminomorpholine, morpholine, dibromoethane, malonyl chloride, and glyoxal (40% aqueous solution) were purchased from Sigma-Aldrich Company and used as received. Acetone and ethanol (96%) were purchased from the Gram-Mol Company and used as received.

Bis­(4-morpholinyl)-1,2-ethane (**1**) was synthesized by dissolving morpholine (2 mmol, 2 equiv) in 5.0 mL of ethanol and adding 1,2-dibromoethane (1 mmol, 1 equiv). The solution was brought to a boil and left undisturbed overnight to evaporate. The resulting slurry was dissolved in an aqueous ammonia solution (10%) and extracted with diethyl ether. The ether fraction was evaporated to give a pale-yellow oil. ^1^H NMR: 2.491 ppm (m, 8H, *J* = 8.581 Hz), 2.711 ppm (s, 4H, *J* = 8.015 Hz), 3.608 ppm (m, 8H, *J* = 11.731 Hz). ^13^C NMR: 51.2 ppm (2C), 53.5 ppm (4C), 66.9 ppm (4C).

Bis­(morpholin-4-yl)­ethane-1,2-diimine (**2**) was synthesized by dissolving 4-aminomorpholine (100 μL) with glyoxal (0.5 equiv) in 5 mL of ethanol with a catalytic amount of concentrated sulfuric acid (5 μL). The solution was left undisturbed and exposed to air for a week, after which light yellow needles of the product appeared. ^1^H NMR: 3.712 ppm, m, 16H, *J* = 10.181 Hz; 7.281 ppm, s, 2H, *J* = 10.158 Hz; ^13^C NMR: 55.8 ppm, 4C; 66.3 ppm, 4C; 156.7 ppm, 2C.

4-Aminomorpholinyl malonamide (**3**) was synthesized by refluxing a mixture of 4-aminomorpholine (40 mmol, 2 equiv) and diethyl malonate (20 mmol, 1 equiv) for 6 h. The solution was left to stand for several days, upon which colorless crystals of the product were obtained. ^1^H NMR: 3.571 ppm (m, 8H, *J* = 15.195 Hz), 3.621 ppm (m, 8H, *J* = 12.018 Hz), 3.718 ppm (s, 2H). ^13^C NMR: 45.2 ppm (1C), 47.3 ppm (4C), 66.7 ppm (4C), 170.1 ppm (2C).

Single crystals were obtained by dissolving the acceptor (∼1 mmol) with an excess of the selected halogen bond donor (∼8 mmol) in ethanol (96%, 5 mL). The large excess of the halogen bond donor was used to maximize the probability of obtaining the stoichiomorphs with the largest number of halogen bonds achieved by the studied acceptor molecules. The obtained solutions were left to evaporate at room temperature until the product crystals would appear, whereupon the crystallization vials were stoppered, and the crystals were left in mother liquor until mounting for single-crystal X-ray diffraction measurements.

In the case of cocrystals of **3** with **1,4-ofib** and **1,4-tfib**, additional crystallization experiments were made. With **1,4-ofib** an attempt was made to produce a hydrate equivalent to **3**_2_·**1,4-tfib**·2H_2_O. To a solution that was prepared earlier, water was added (up to ca. 0.5 mL—larger amounts led to separation of **1,4-ofib**), and the solution was left to evaporate at room temperature. This yielded crystals that were found to be identical to the previously obtained **3**_2_·**1,4-ofib**. With **1,4-tfib**, an attempt was made to produce a nonsolvated cocrystal. A solution prepared as above was dried over zeolite molecular sieves, filtered, and left to evaporate in a desiccator over sodium hydroxide. The isolated crystals were found to be the hydrate **3**_2_·**1,4-tfib**·2H_2_O and pure **3**.

### Single-Crystal X-ray Diffraction

Single-crystal X-ray diffraction data were collected on an Oxford Diffraction Xcalibur3 CCD diffractometer with graphite-monochromatized Mo Kα radiation (λ = 0.71073 Å). Room-temperature single-crystal X-ray diffraction data were collected on an XtaLAB Synergy-S diffractometer using Mo Kα radiation. The data sets were collected by using the ω scan mode over the 2θ range up to 64° (Synergy XtaLAB). Programs CrysAlis CCD, CrysAlis RED, and CrysAlisPro^42^ were employed for data collection, cell refinement, and data reduction. All structures were solved by direct methods (SHELXS)^43^ for the disordered structures and by dual-space methods (SHELXT)^44^ for the rest in the Olex2 suite,^45^ and the refinement procedure was performed by the full-matrix least-squares method based on all reflections using SHELXL.^46^ The non-hydrogen atoms were refined anisotropically.^43–46^ The crystal structure of **2**·H_2_O was refined as a twin with the twin law [−1 0 −1.333 0 1 0 0 0 1] and 15.7% fractional contribution of the second component.

All aliphatic hydrogen atoms were placed in calculated positions and refined using the riding model. All hydrogen atoms bonded to oxygen or nitrogen atoms were found in difference Fourier maps except for the hydrogen atoms of the water molecules in structures **2**_2_·**1,4-tfib**·2H_2_O and **2**·H_2_O, which were modeled by fixing the O–H distances to 0.85(1) Å and the H–H distances to 1.39(2) Å. Drawings of the structures were prepared using Mercury^47^ and Olex2 programs.

The crystallographic data are summarized in Tables S1–S3. Further details are available from the Cambridge Crystallographic Centre (CCDC 2442787–2442798 and CCDC 2444049 contain crystallographic data for this paper).

### Computational Studies

All calculations were performed using the Gaussian 16 software package.^48^ Geometry optimizations were performed using the M06-2X/def2-tzvp level of theory^49^ with an ultrafine integration grid (99 radial shells and 590 points per shell). It has been shown that this functional in combination with the triple-ζ basis set provides quite accurate geometries of halogen-bonded molecular complexes as well as their energies.^50,51^ Harmonic frequency calculations were performed on the optimized geometries to ensure the success of each geometry optimization. The figures were prepared using GaussView.^52^

## Supplementary Material



## Notes

Additional experimental details pertaining to the preparation of the morpholine derivatives **1**–**3**, crystal data, refinement details, ORTEP plots of asymmetric units and selected bond lengths for all studied crystals and prepared cocrystals, and statistical comparison of halogen bond parameters observed in this study to those in the CSD (PDF)
