# Supplementary material for: Morpholine Nitrogen and Oxygen Atoms as Halogen Bond Acceptors in Different Environments: Cocrystals of Amino‑, Imino- or Amide-Linked Bis(morpholine) Derivatives with Perfluorinated Iodohydrocarbons
Source: Cryst Growth Des. 2026 Jun 18;26(13):5033–41. doi: 10.1021/acs.cgd.6c00316 (PMC13741371; doi:10.1021/acs.cgd.6c00316)
Supplement: Supplementary file 1 [file cg6c00316_si_001.pdf]

Morpholine Nitrogen and Oxygen Atoms as Halogen Bond Acceptors in Different Environments – Cocrystals of Amino-, Imino- or Amide-Linked bis-Morpholinyl Derivatives with with Perfluorinated Iodohydrocarbons

*Kristina Smoković,[a,b] Nikola Bedeković [b] Vladimir Stilinović[b]**

[a] Jožef Stefan Institute, Jamova cesta 39, 1000 Ljubljana, Slovenia

[b] Department of Chemistry, Faculty of Science, University of Zagreb, Horvatovac 102a, 10000 Zagreb, Croatia.

Electronic Supplementary Information

Contents

Contents 2

1. Synthesis of morpholine derivatives 3
2. Crystallographic information 4
3. Halogen bond parameter comparisons 19

1. Synthesis of morpholine derivatives

All halogen bond donors (1,2,3,4-tetrafluoro-5,6-diiodobenzene, 1,2,3,5-tetrafluoro-4,6-diiodobenzene, 1,2,4,5-tetrafluoro-3,6-diiodobenzene, 1,3,5-trifluoro-2,4,6-triiodobenzene, and 1,1,2,2,3,3,4,4-octafluoro-1,4-diiodobutane) were purchased from Manchester Organics Company; *N*-aminomorpholine, morpholine, dibromoethane, malonyl chloride and glyoxal (40% aqueous solution) were purchased from Sigma-Aldrich Company and used as received. Acetone and ethanol (96%) were purchased from Gram-Mol company and used as received.

Bis(4-morpholinyl)-1,2-ethane (**1**) was synthesized by dissolving morpholine (2 mmol, 2 eqv.) in 5,0 mL of ethanol and adding 1,2-dibromoethane (1 mmol, 1 eqv.). The solution was brought to a boil and left undisturbed overnight to evaporate. 4-Aminomorpholinyl malonamide (**2**) was synthesized by refluxing a mixture of 4-aminomorpholine (40 mmol, 2 eqv.) and diethyl malonate (20 mmol, 1 eqv.) for 6 hours. The solution was left to stand for several days, after which crystals appeared. Bis(morpholin-4-yl)ethane-1,2-diimine (**3**) was synthesized by dissolving 4-aminomorpholine (100 µL) with glyoxal (0.5 eq.) in 5 mL of ethanol with a catalytic amount of concentrated sulfuric acid (5 µL). The solution was left undisturbed and exposed to air for a week, after which light yellow needles of the product appeared.

2. Crystallographic information

Table 1. Crystal data and refinement details for the prepared cocrystals of bis(4-morpholinyl)-1,2-ethane (**1**) with halogen bond donors.

Sample	**1**·12**tfib**$_2$	**1**·13tfib	**1**·14**tfib**· **1**	**1**·135**tfib**$_4$
Empirical formula	$C_{11}H_{10}F_4I_2NO$	$C_{16}H_{18}F_4I_2N_2O_2$	$C_{26}H_{40}F_4I_2N_4O_4$	$C_{34}H_{20}F_{12}I_{12}N_2O_2$
MW/gmol^{-1}	502.00	600.12	802.42	2239.32
T/K	298	298	298	293
Crystal system	monoclinic	monoclinic	triclinic	triclinic
Space group	$I2/m$	$I2/a$	$P\bar{1}$	$P\bar{1}$
a/Å	9.5313(5)	7.2071(3)	6.1037(4)	9.4413(11)
b/Å	7.4985(5)	17.1429(6)	10.3816(5)	9.4544(11)
c/Å	21.2509(12)	16.5246(5)	13.6640(6)	14.3972(16)
α/°	90	90	102.105(4)	97.959(9)
β/°	99.872(6)	94.005(3)	102.414(5)	104.007(10)
γ/°	90	90	105.887(5)	96.085(9)
V/Å^3	1496.32(15)	2036.64(13)	779.35(8)	1221.8(3)
Z	4	4	1	1
ρ_{calc} /gcm^{-3}	2.228	1.957	1.71	3.043
μ/mm^{-1}	4.237	3.136	2.078	7.684
F(000)	932	1144	398	998
λ (MoKα)/ Å	0.71073	0.71073	0.71073	0.71073
2Θ range for data collection/°	8.886 - 53.986	9.112 - 53.994	8.62 - 53.998	8.706 - 53.994
Index ranges	$-10 \le h \le 12$, $-9 \le k \le 8$, $-27 \le l \le 22$	$-9 \le h \le 9$, $-17 \le k \le 21$, $-9 \le l \le 21$	$-7 \le h \le 7$, $-13 \le k \le 13$, $-17 \le l \le 17$	$-7 \le h \le 12$, $-12 \le k \le 11$, $-18 \le l \le 18$
Reflections collected	3574	4675	6441	10168
Independent reflections	R_{int} = 0.0196, R_{sigma} = 0.0265	R_{int} = 0.0260, R_{sigma} = 0.0352	R_{int} = 0.0298, R_{sigma} = 0.0356	R_{int} = 0.0433, R_{sigma} = 0.0637
Data/restraints/parameters	1757/0/113	2206/0/130	3392/0/182	5302/0/281
Goodness-of-fit on F^2	1.027	1.046	1.073	1.105
Final R indexes [I>=2σ (I)]	R_1 = 0.0270, wR_2 = 0.0597	R_1 = 0.0313, wR_2 = 0.0702	R_1 = 0.0255, wR_2 = 0.0569	R_1 = 0.0551, wR_2 = 0.1197
Final R indexes [all data]	R_1 = 0.0350, wR_2 = 0.0645	R_1 = 0.0422, wR_2 = 0.0791	R_1 = 0.0303, wR_2 = 0.0592	R_1 = 0.0788, wR_2 = 0.1313
Dr_{max}/Dr_{min} / e Å^{-3}	0.50/−0.57	0.51/−0.77	0.84/−0.60	1.56/−1.53

Table 2. Crystal data and refinement details 4-aminomorpholinyl malondiamide (**2**) and for the prepared cocrystals with halogen bond donors.

Sample	**2**	$\mathbf{2}_2$·14**tfib**· 2 H_2O	**2**·135**tfib**	$\mathbf{2}_2$·14**ofib**
Empirical formula	$C_{22}H_{36}N_4O_8$	$C_{14}H_{20}F_2IN_2O_5$	$C_{17}H_{18}F_3I_3N_2O_4$	$C_{13}H_{18}F_4IN_2O_4$
MW/gmol^{-1}	484.55	461.22	752.03	469.19
T/K	298	293	298	298
Crystal system	triclinic	monoclinic	monoclinic	monoclinic
Space group	$P\overline{1}$	$P2_1/c$	$P2_1/c$	$P2_1/c$
a/Å	5.2905(13)	27.8386(14)	7.1737(6)	16.201(2)
b/Å	9.916(2)	7.1585(5)	9.1781(6)	5.6409(6)
c/Å	11.106(3)	9.0341(5)	32.947(2)	19.284(2)
α/°	86.215(19)	90	90	90
β/°	88.255(19)	97.406(5)	92.118(7)	102.865(13)
γ/°	84.18(2)	90	90	90
V/Å^3	578.2(2)	1785.32(18)	2167.7(3)	1718.1(4)
Z	1	4	4	4
ρ_{calc} /gcm^{-3}	1.392	1.716	2.304	1.814
μ/mm^{-1}	0.106	1.839	4.377	1.924
F(000)	260	916	1408	924
λ (MoKα)/ Å	0.71073	0.71073	0.71073	0.71073
2Θ range for data collection/°	8.668 - 53.998	8.828 - 57.998	8.63 - 53.998	8.426 - 51.996
Index ranges	$-6 \leq h \leq 6$, $-7 \leq k \leq 12$, $-14 \leq l \leq 13$	$-37 \leq h \leq 37$, $-9 \leq k \leq 4$, $-12 \leq l \leq 12$	$-9 \leq h \leq 9$, $-11 \leq k \leq 11$, $-42 \leq l \leq 42$	$-19 \leq h \leq 19$, $-6 \leq k \leq 6$, $-23 \leq l \leq 23$
Reflections collected	3891	11401	26795	6870
Independent reflections	R_{int} = 0.0786, R_{sigma} = 0.0947	R_{int} = 0.0403, R_{sigma} = 0.0600	R_{int} = 0.0696, R_{sigma} = 0.0626	R_{int} = 0.0691, R_{sigma} = 0.1020
Data/restraints/parameters	2481/0/154	4647/0/221	4704/0/262	3337/0/217
Goodness-of-fit on F^2	0.998	0.987	1.01	1.035
Final R indexes [I>=2σ (I)]	R_1 = 0.0636, wR_2 = 0.1353	R_1 = 0.0397, wR_2 = 0.0638	R_1 = 0.0431, wR_2 = 0.0567	R_1 = 0.0700, wR_2 = 0.0815
Final R indexes [all data]	R_1 = 0.1148, wR_2 = 0.1631	R_1 = 0.0969, wR_2 = 0.0783	R_1 = 0.0687, wR_2 = 0.0625	R_1 = 0.1441, wR_2 = 0.0978
Dr_{max}/Dr_{min} / e Å^{-3}	0.30/−0.23	0.47/−0.46	0.90/−0.61	0.53/−0.48

Table 3. Crystal data and refinement details for the prepared cocrystals of bis(4-morpholinyl)-1,2-ethane (1) with halogen bond donors

Sample	**3**·12**tfib**$_2$	**3**·13**tfib**$_2$	**3**·14**tfib**$_2$	**3**·135**tfib**$_2$
Empirical formula	$C_{22}H_{15}F_8I_4N_4O_2$	$C_{22}H_{18}F_8I_4N_4O_2$	$C_{22}H_{18}F_8I_4N_4O_2$	$C_{44}H_{36}F_{12}I_{12}N_8O_4$
MW/gmol^{-1}	515	1030	1030.00	2491.61
T/K	293(2)	292.99(13)	298	293(2)
Crystal system	monoclinic	triclinic	triclinic	triclinic
Space group	$P2_1$/n	$P\bar{1}$	$P\bar{1}$	$P\bar{1}$
a/Å	4.8531(2)	4.47910(10)	7.0729(7)	9.2906(8)
b/Å	28.8812(13)	12.9910(9)	7.1653(7)	12.0414(10)
c/Å	10.8103(4)	13.2904(4)	16.0351(16)	14.8436(12)
α/°	90	81.376(4)	86.668(8)	86.014(7)
β/°	102.130(4)	81.098(3)	89.819(8)	84.403(7)
γ/°	90	82.386(4)	69.777(9)	75.727(7)
V/Å^3	1481.38(11)	750.64(6)	761.14(14)	1599.9(2)
Z	4	1	1	1
ρ_{calc} /gcm^{-3}	2.309	2.279	2.247	2.586
μ/mm^{-1}	4.285	4.228	4.17	5.888
F(000)	956	478	478	1132
λ (MoKα)/ Å	0.71073	0.71073	0.71073	0.71073
2Θ range for data collection/°	8.646 - 75.324	6.26 - 53.994	8.796 - 53.996	8.706 - 53.998
Index ranges	$-7 \le h \le 8$, $-45 \le k \le 44$, $-14 \le l \le 17$	$-5 \le h \le 5$, $-16 \le k \le 16$, $0 \le l \le 16$	$-9 \le h \le 8$, $-8 \le k \le 9$, $-19 \le l \le 20$	$-11 \le h \le 9$, $-15 \le k \le 14$, $-18 \le l \le 18$
Reflections collected	18490	3261	5860	10944
Independent reflections	R_{int} = 0.0593, R_{sigma} = 0.0789	R_{int} = 0.0787, R_{sigma} = 0.0718	R_{int} = 0.0391, R_{sigma} = 0.0534	R_{int} = 0.0462, R_{sigma} = 0.0922
Data/restraints/parameters	6816/12/211	3261/0/183	3285/0/182	6862/0/362
Goodness-of-fit on F^2	0.928	1.113	0.987	0.96
Final R indexes [I>=2σ (I)]	R_1 = 0.0618, wR_2 = 0.1481	R_1 = 0.0629, wR_2 = 0.2257	R_1 = 0.0399, wR_2 = 0.0833	R_1 = 0.0566, wR_2 = 0.1021
Final R indexes [all data]	R_1 = 0.1496, wR_2 = 0.2034	R_1 = 0.0789, wR_2 = 0.2465	R_1 = 0.0542, wR_2 = 0.0903	R_1 = 0.1078, wR_2 = 0.1246
Dr_{max}/Dr_{min} / e Å^{-3}	0.84/−2.21	1.68/−1.00	0.85/−1.04	1.08/−1.05

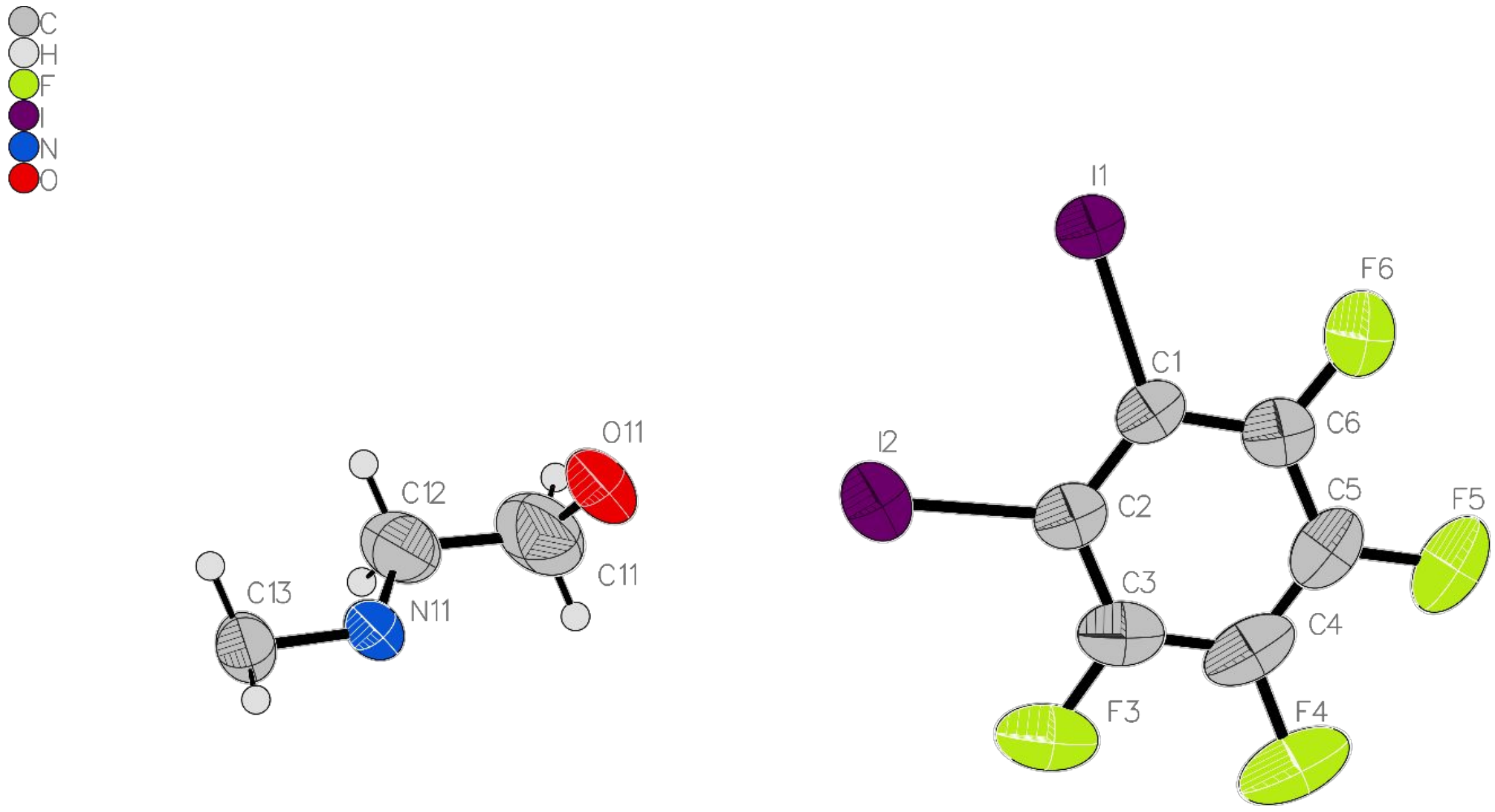

Figure 1 Asymetric unit of **1**·12**tfib**$_2$ showing the atom-labelling scheme. Displacement ellipsoids are drawn at the 50 % probability level, and H atoms are shown as small spheres of arbitrary radius.

Table 4 Bond Lengths for **1**·12**tfib**$_2$.

**Atom (A1)**	**Atom (A2)**	***d*(A1 − A2)/Å**	**Atom (A1)**	**Atom (A2)**	***d*(A1 − A2)/Å**
I1	C1	2.085(4)	N11	C12^1	1.450(5)
I2	C2	2.081(5)	N11	C12	1.450(5)
F6	C6	1.346(6)	N11	C13^1	1.552(7)
F3	C3	1.341(7)	N11	C13	1.552(7)
F4	C4	1.335(6)	C2	C3	1.377(7)
F5	C5	1.343(6)	C6	C5	1.346(7)
O11	C11^1	1.407(5)	C5	C4	1.342(8)
O11	C11	1.407(5)	C3	C4	1.386(8)
C1	C2	1.385(6)	C11	C12	1.490(6)
C1	C6	1.391(7)	C13	C13^2	1.508(12)

^{1}+*x*, 1-*y*, +*z*; ^{2}2−*x*, 1−*y*, 1−*z*

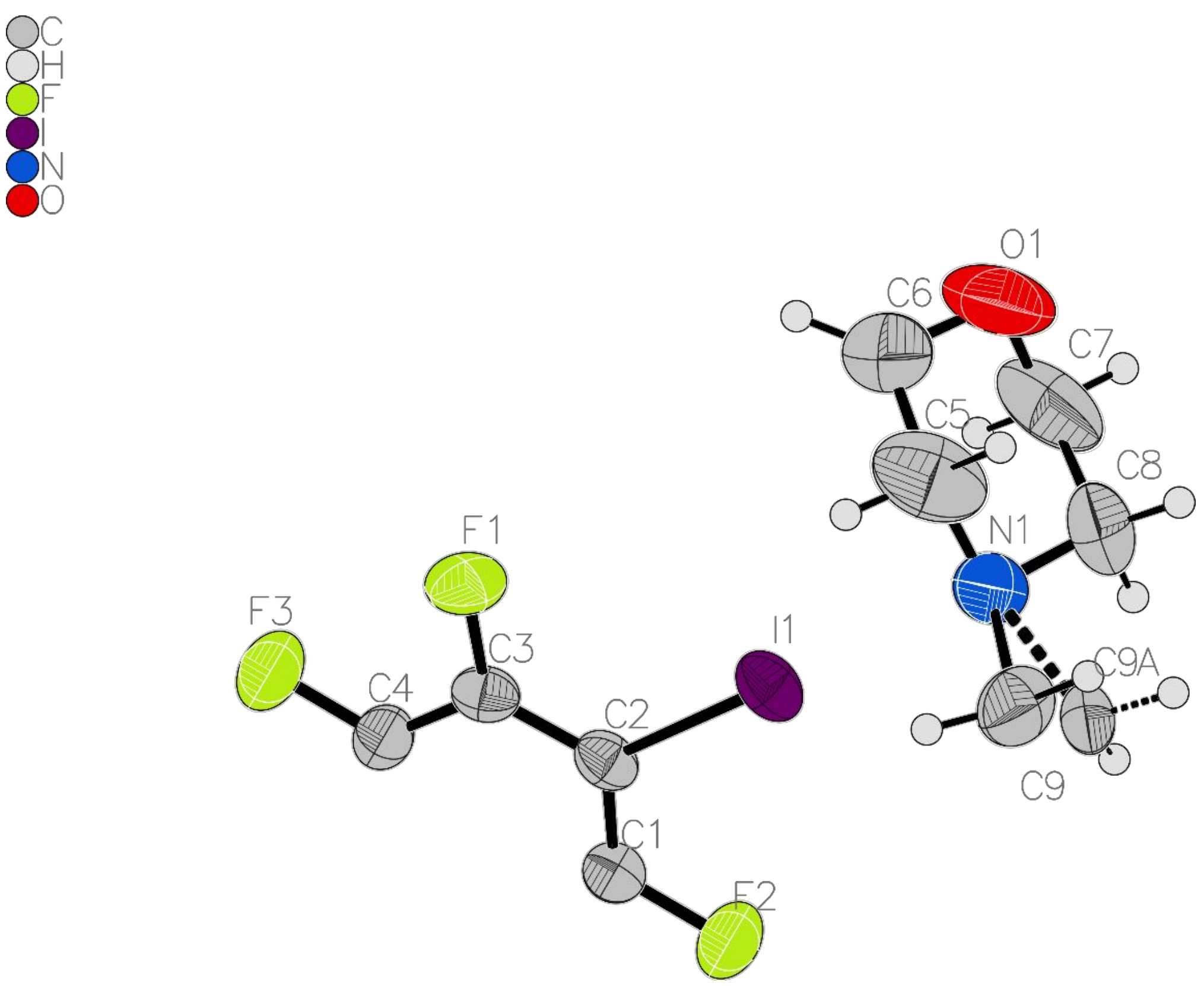

Figure 2 Asymetric unit of **1**·13**tfib** showing the atom-labelling scheme. Displacement ellipsoids are drawn at the 50 % probability level, and H atoms are shown as small spheres of arbitrary radius.

Table 5 Bond Lengths for **1**·13**tfib**.

Atom (A1)	Atom (A2)	*d*(A1 − A2)/Å	Atom (A1)	Atom (A2)	*d*(A1 − A2)/Å
I1	C2	2.100(4)	N1	C5	1.471(7)
F1	C3	1.336(4)	N1	C8	1.440(8)
F2	C1	1.358(6)	N1	C9	1.519(10)
F3	C4	1.334(6)	N1	C9A	1.557(18)
C2	C3	1.377(5)	C6	C5	1.475(9)
C2	C1	1.370(4)	C7	C8	1.501(9)
C3	C4	1.375(4)	C9	C9[1]	1.510(15)
O1	C6	1.378(8)	C9A	C9A[1]	1.52(3)
O1	C7	1.431(9)			

[1]1/2−*x*, 3/2−*y*, 1/2−*z*

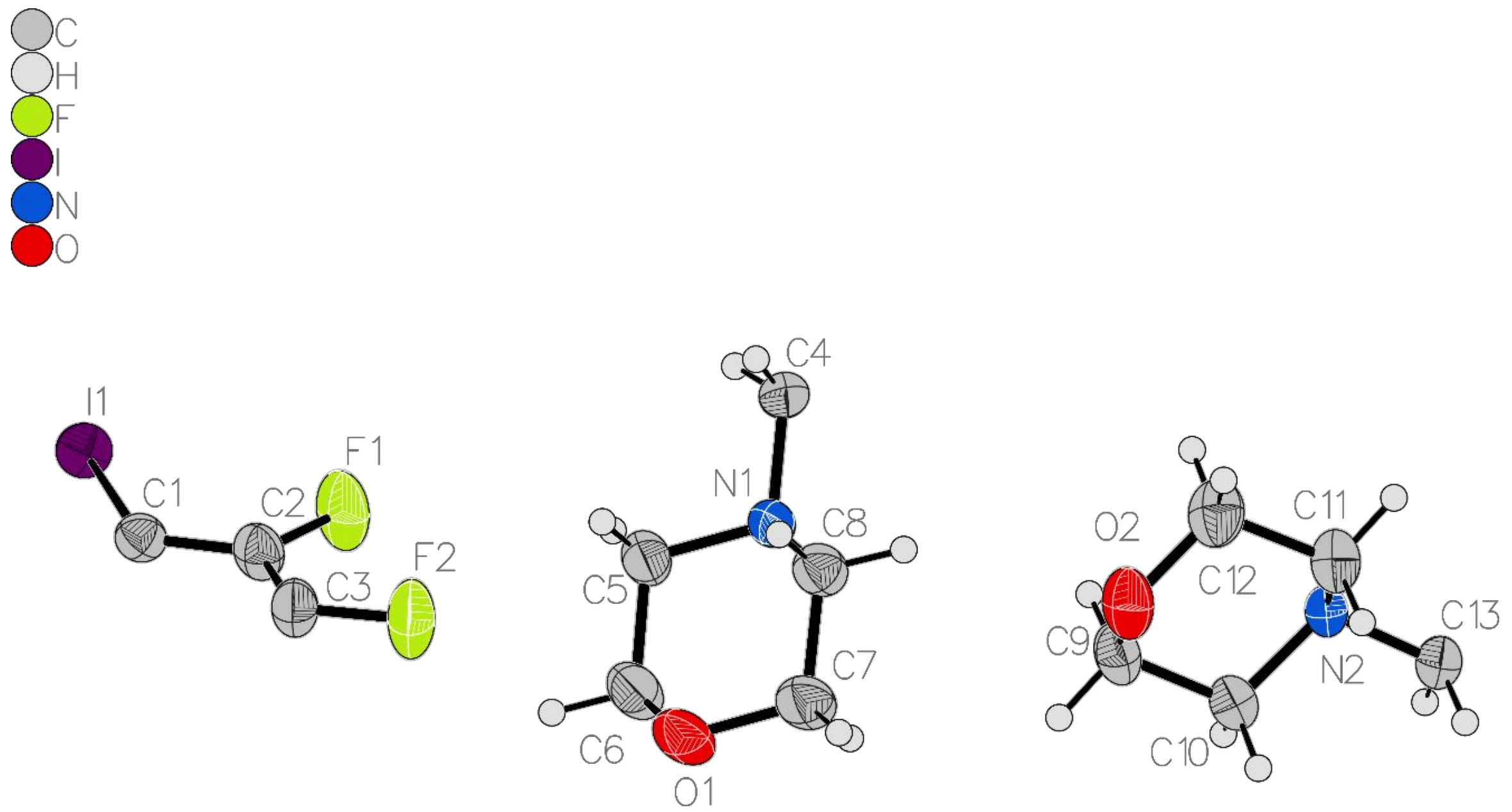

Figure 3 Asymetric unit of **1**·14**tfib**· **1** showing the atom-labelling scheme. Displacement ellipsoids are drawn at the 50 % probability level, and H atoms are shown as small spheres of arbitrary radius.

Table 6 Bond Lengths for **1**·14**tfib**· **1**.

**Atom (A1)**	**Atom (A2)**	***d*(A1 − A2)/Å**	**Atom (A1)**	**Atom (A2)**	***d*(A1 − A2)/Å**
I1	C1	2.104(3)	N2	C10	1.467(3)
F2	C3	1.340(3)	N2	C13	1.470(3)
F1	C2	1.348(3)	C1	$C3^1$	1.382(3)
O2	C12	1.421(4)	C1	C2	1.374(4)
O2	C9	1.418(3)	C3	C2	1.372(4)
O1	C6	1.415(4)	C11	C12	1.514(4)
O1	C7	1.430(4)	C10	C9	1.502(4)
N1	C4	1.454(3)	C4	$C4^2$	1.532(5)
N1	C8	1.454(3)	C8	C7	1.503(4)
N1	C5	1.466(3)	C13	$C13^3$	1.509(5)
N2	C11	1.467(3)	C6	C5	1.500(4)

^{1}−1−*x*, 1−*y*, −1−*z*; ^{2}1−*x*, 1−*y*, −*z*; ^{3}3−*x*, 2−*y*, 1−*z*

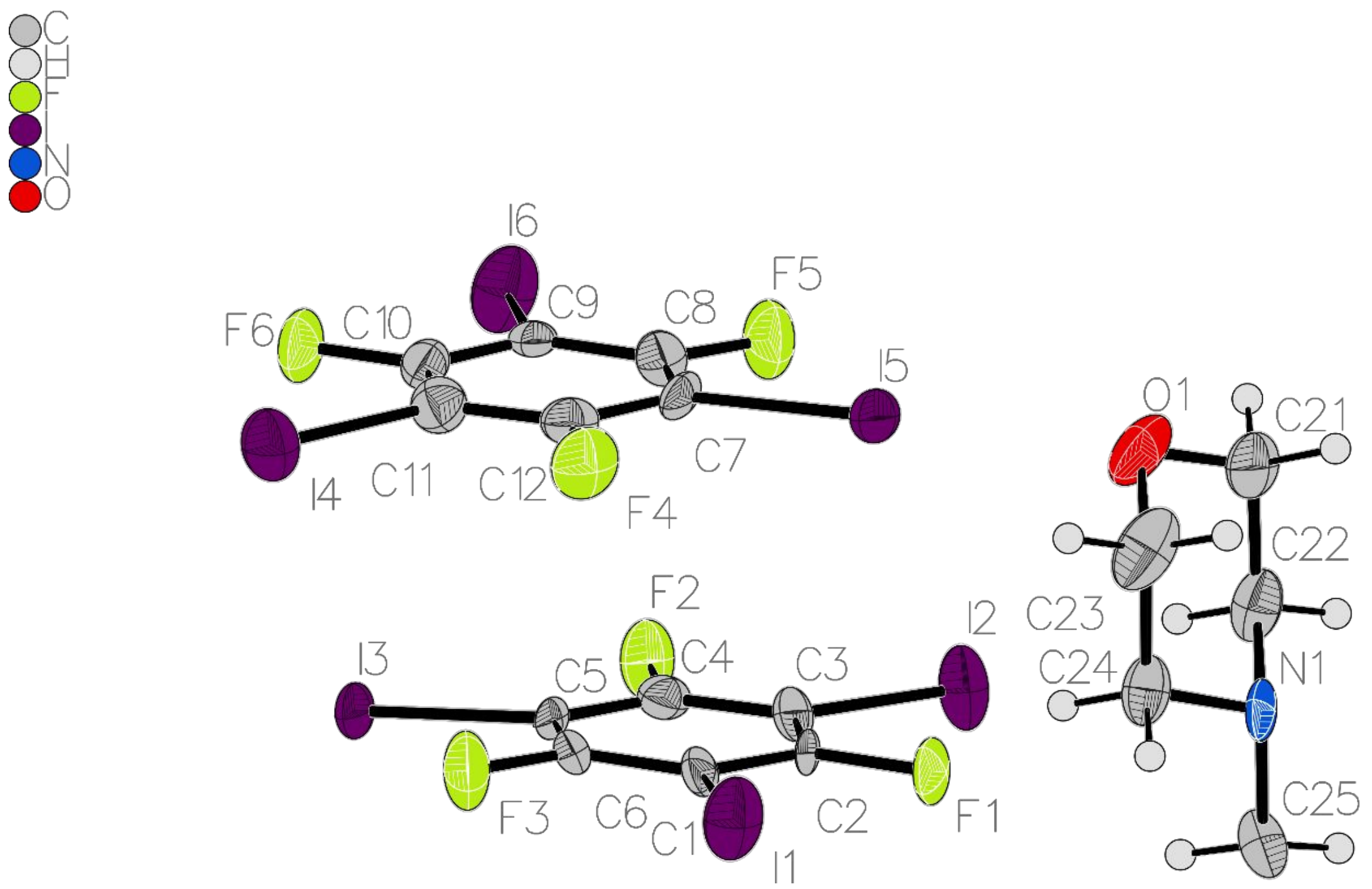

Figure 4 Asymetric unit of **1**·135**tfib**$_4$ showing the atom-labelling scheme. Displacement ellipsoids are drawn at the 50 % probability level, and H atoms are shown as small spheres of arbitrary radius.

Table 7 Bond lengths for **1**·135**tfib**$_4$.

**Atom (A1)**	**Atom (A2)**	***d*(A1 − A2)/Å**	**Atom (A1)**	**Atom (A2)**	***d*(A1 − A2)/Å**
I3	C5	2.110(7)	N1	C24	1.449(9)
I5	C7	2.096(7)	C3	C2	1.368(11)
I4	C11	2.062(8)	C3	C4	1.368(11)
I2	C3	2.057(8)	C2	C1	1.370(10)
I1	C1	2.067(8)	C6	C5	1.343(12)
I6	C9	2.065(9)	C6	C1	1.389(10)
F1	C2	1.335(8)	C10	C11	1.369(12)
F3	C6	1.335(9)	C10	C9	1.357(12)
F6	C10	1.335(9)	C12	C7	1.362(12)
F5	C8	1.334(10)	C12	C11	1.395(11)
F4	C12	1.317(10)	C5	C4	1.372(11)
F2	C4	1.320(10)	C7	C8	1.348(13)
O1	C23	1.423(13)	C25	C25[1]	1.498(18)
O1	C21	1.412(12)	C22	C21	1.502(14)
N1	C25	1.456(11)	C9	C8	1.396(11)
N1	C22	1.458(12)	C24	C23	1.508(15)

[1]3−*x*, 3−*y*, 1−*z*

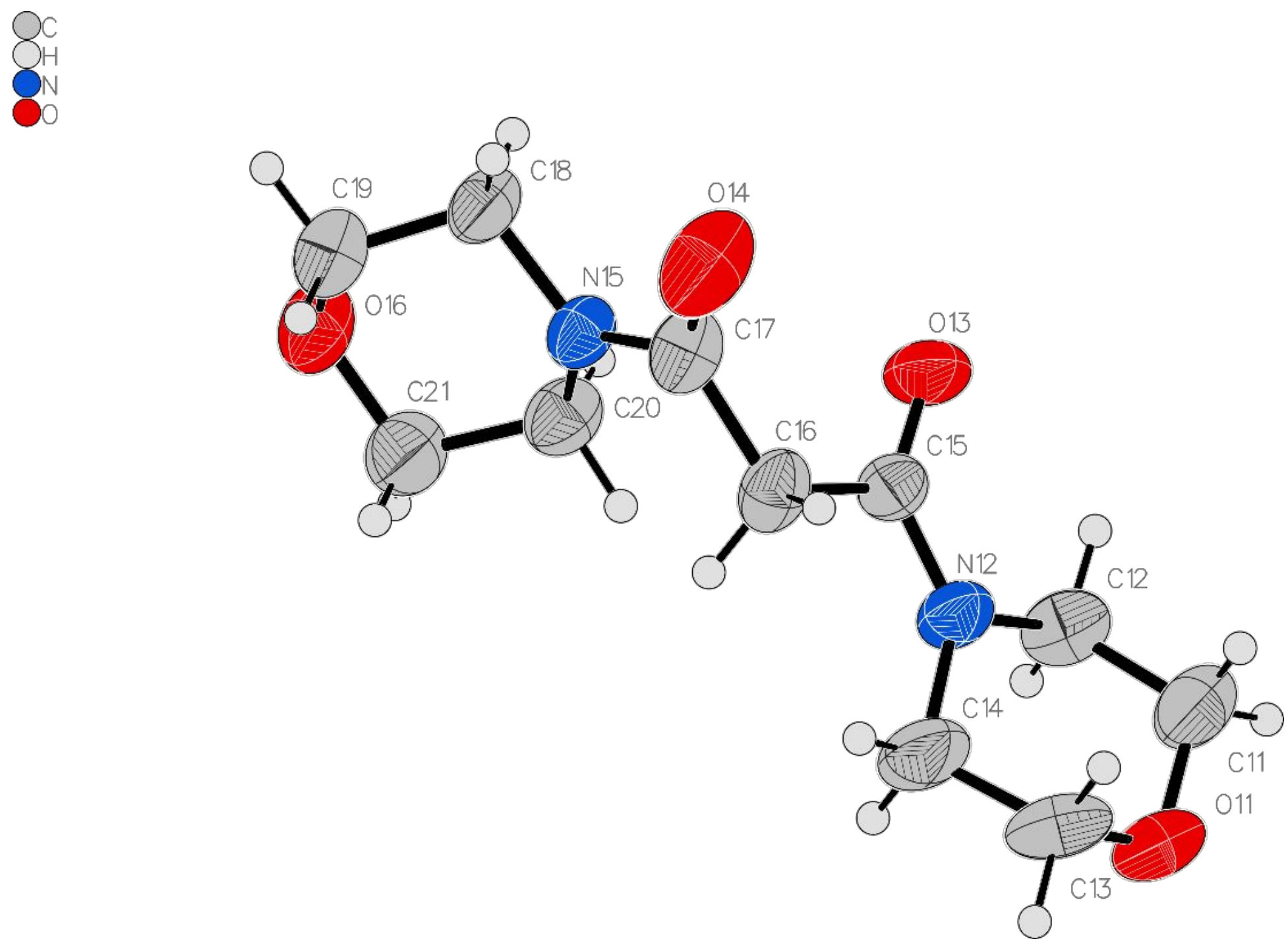

Figure 5 Asymetric unit of **2** showing the atom-labelling scheme. Displacement ellipsoids are drawn at the 50 % probability level, and H atoms are shown as small spheres of arbitrary radius.

Table 8 Bond lengths for **2**

**Atom (A1)**	**Atom (A2)**	***d*(A1 − A2)/Å**	**Atom (A1)**	**Atom (A2)**	***d*(A1 − A2)/Å**
O13	C15	1.210(3)	N12	C15	1.329(3)
O16	C19	1.408(3)	N12	C12	1.426(3)
O16	C21	1.416(3)	N12	C14	1.451(4)
N15	C17	1.322(3)	C15	C16	1.500(3)
N15	C18	1.454(3)	C17	C16	1.507(4)
N15	C20	1.442(3)	C18	C19	1.484(4)
O11	C13	1.387(3)	C20	C21	1.485(4)
O11	C11	1.405(4)	C12	C11	1.481(4)
O14	C17	1.210(3)	C13	C14	1.467(4)

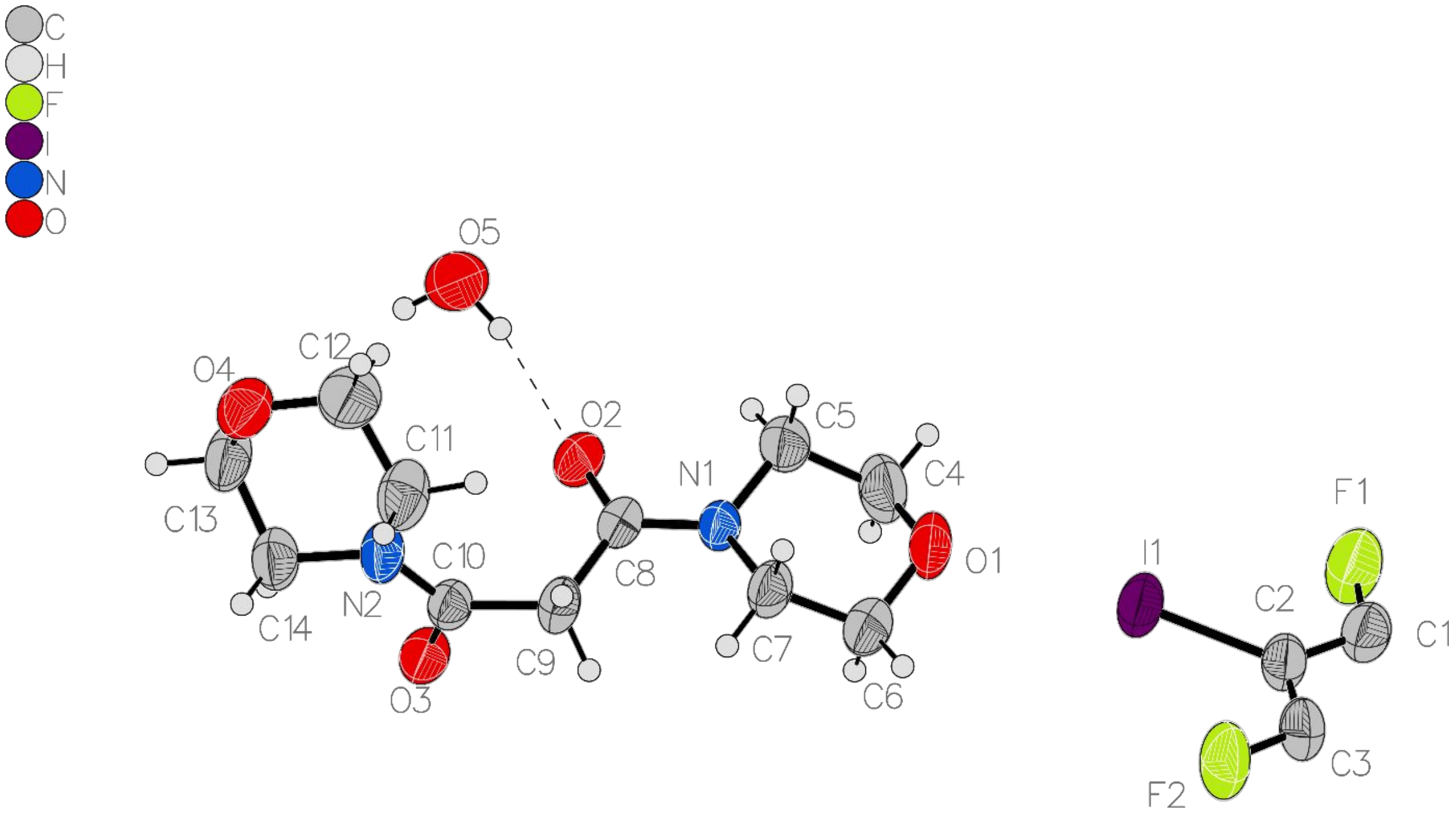

Figure 6 Asymetric unit of $\mathbf{2}_2$·14**tfib** · 2 H_2O showing the atom-labelling scheme. Displacement ellipsoids are drawn at the 50 % probability level, and H atoms are shown as small spheres of arbitrary radius.

Table 9 Bond lengths for $\mathbf{2}_2$·14**tfib** · 2 H_2O.

Atom (A1)	Atom (A2)	*d*(A1 − A2)/Å	Atom (A1)	Atom (A2)	*d*(A1 − A2)/Å
I1	C1	2.081(3)	N12	C12	1.465(4)
O13	C15	1.225(4)	N12	C14	1.456(4)
O14	C17	1.227(4)	C17	C16	1.517(5)
F2	C2	1.346(4)	C15	C16	1.524(4)
O11	C11	1.415(4)	C1	C2	1.364(5)
O11	C13	1.415(4)	C1	C3	1.385(5)
F3	C3	1.341(4)	C18	C19	1.492(4)
N15	C17	1.340(4)	C12	C11	1.484(5)
N15	C18	1.466(4)	C2	$C3^1$	1.371(5)
N15	C20	1.470(4)	C3	$C2^1$	1.371(5)
O16	C19	1.417(4)	C14	C13	1.475(5)
O16	C21	1.405(5)	C20	C21	1.495(5)

[1] $1-x$, $1-y$, $-z$

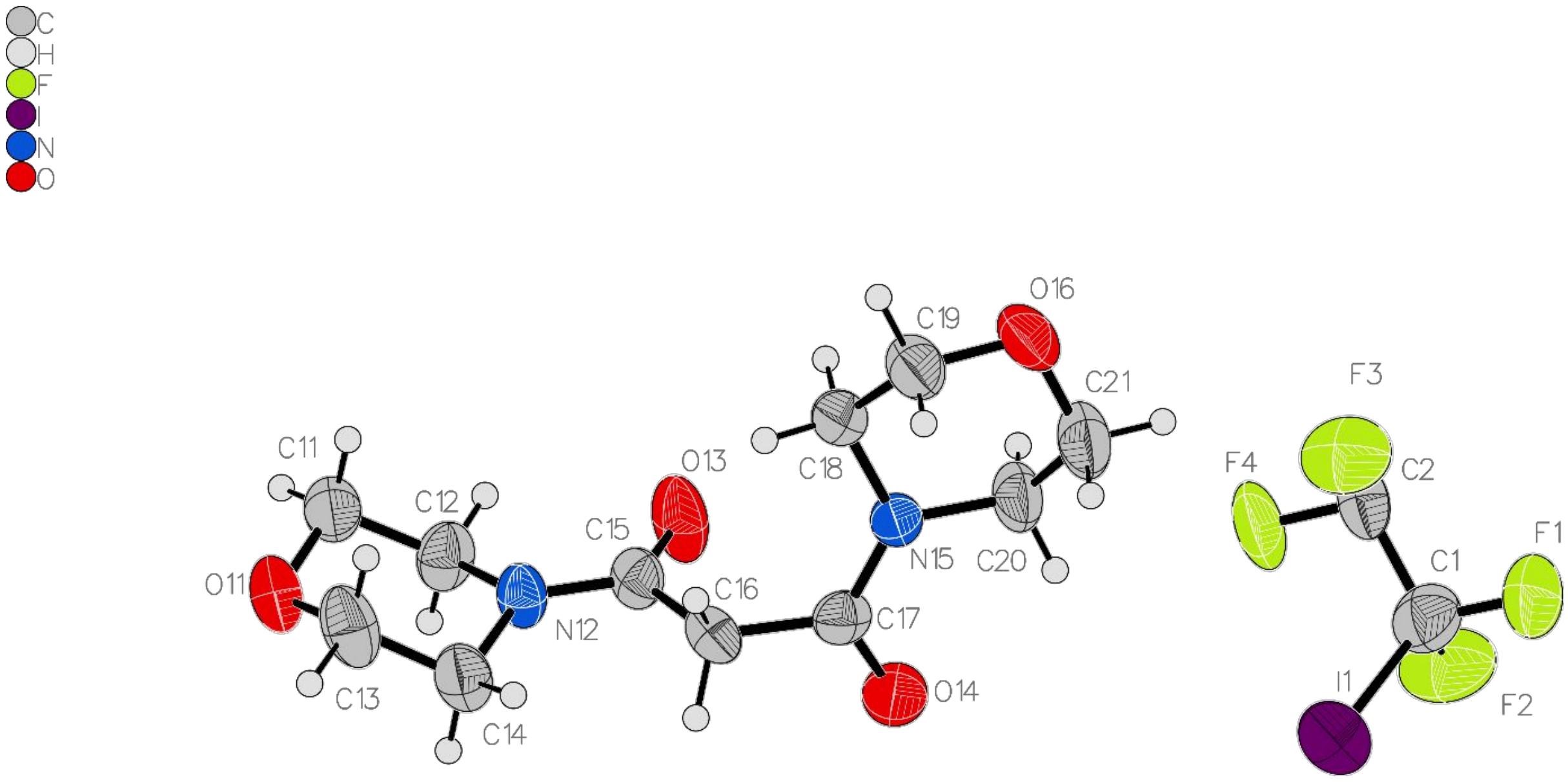

Figure 7 Asymetric unit of $\mathbf{2}_2$·14**ofib** showing the atom-labelling scheme. Displacement ellipsoids are drawn at the 50 % probability level, and H atoms are shown as small spheres of arbitrary radius.

Table 10 Bond lengths for $\mathbf{2}_2$·14ofib.

**Atom (A1)**	**Atom (A2)**	***d*(A1 − A2)/Å**	**Atom (A1)**	**Atom (A2)**	***d*(A1 − A2)/Å**
I1	C1	2.128(8)	N12	C15	1.331(8)
F4	C2	1.332(8)	N12	C14	1.445(8)
O14	C17	1.219(6)	N12	C12	1.456(7)
O16	C21	1.408(7)	F3	C2	1.335(7)
O16	C19	1.420(8)	C15	C16	1.502(8)
O13	C15	1.210(7)	C16	C17	1.512(8)
N15	C17	1.338(8)	C21	C20	1.496(8)
N15	C18	1.457(7)	C18	C19	1.489(9)
N15	C20	1.462(7)	C14	C13	1.497(8)
F2	C1	1.323(8)	C12	C11	1.498(7)
F1	C1	1.342(8)	C2	C2[1]	1.553(14)
O11	C13	1.418(7)	C2	C1	1.528(9)
O11	C11	1.412(8)			

[1]−*x*, 1−*y*, 1−*z*

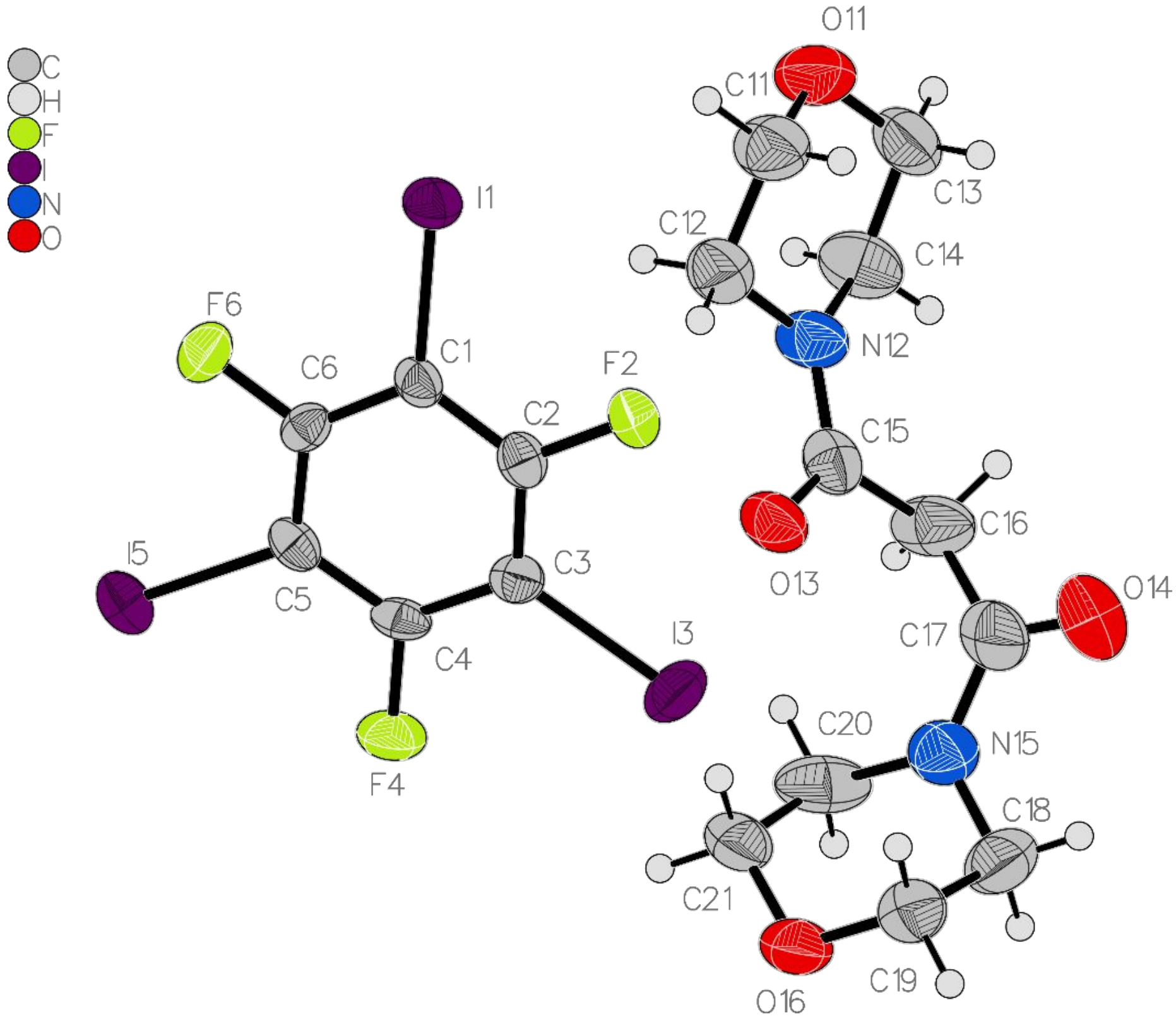

Figure 8 Asymetric unit of **2**·135**tfib** showing the atom-labelling scheme. Displacement ellipsoids are drawn at the 50 % probability level, and H atoms are shown as small spheres of arbitrary radius.

Table 11 Bond lengths for **2**·135**tfib**.

**Atom (A1)**	**Atom (A2)**	***d*(A1 − A2)/Å**	**Atom (A1)**	**Atom (A2)**	***d*(A1 − A2)/Å**
I1	C1	2.075(5)	O11	C13	1.406(7)
I5	C5	2.082(5)	O11	C11	1.425(7)
I3	C3	2.071(5)	N12	C15	1.333(7)
F2	C2	1.345(6)	N12	C14	1.458(7)
F4	C4	1.340(5)	N12	C12	1.460(7)
F6	C6	1.339(5)	N15	C17	1.356(8)
O13	C15	1.232(6)	N15	C18	1.471(8)
O16	C19	1.406(7)	N15	C20	1.438(8)
O16	C21	1.417(7)	O14	C17	1.227(9)
C5	C6	1.371(7)	C15	C16	1.515(8)
C5	C4	1.378(7)	C19	C18	1.486(8)
C3	C2	1.370(7)	C13	C14	1.480(8)
C3	C4	1.380(7)	C17	C16	1.510(9)
C1	C6	1.386(7)	C12	C11	1.474(8)
C1	C2	1.380(7)	C21	C20	1.513(9)

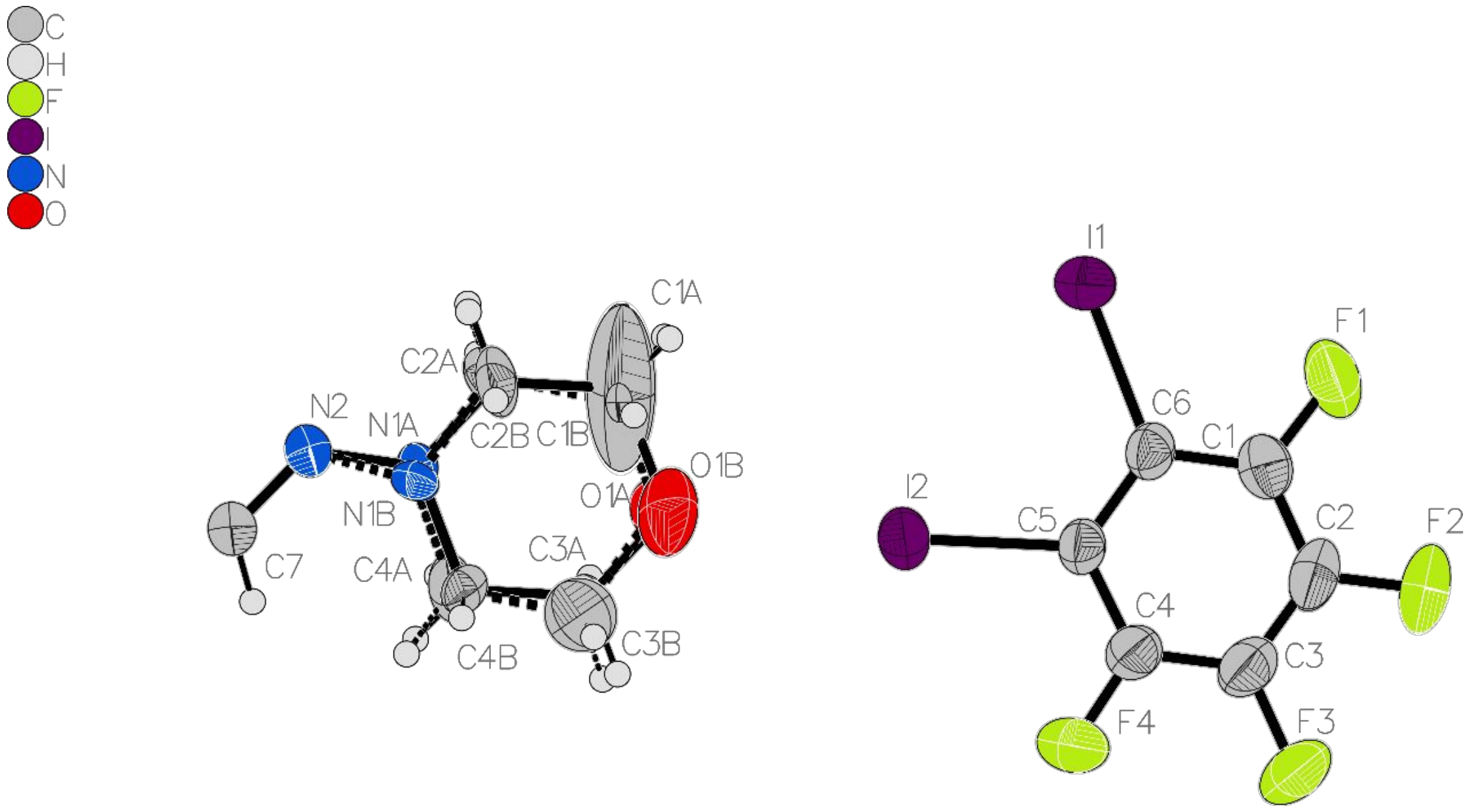

Figure 9 Asymetric unit of **3**·12**tfib**$_2$ showing the atom-labelling scheme. Displacement ellipsoids are drawn at the 50 % probability level, and H atoms are shown as small spheres of arbitrary radius.

Table 12 Bond lenghts for **3**·12**tfib**$_2$.

**Atom (A1)**	**Atom (A2)**	***d*(A1 − A2)/Å**	**Atom (A1)**	**Atom (A2)**	***d*(A1 − A2)/Å**
I2	C5	2.080(5)	C1	C2	1.380(10)
I1	C6	2.092(6)	C3	C2	1.366(10)
F4	C4	1.336(8)	C1B	O1B	1.5381
F1	C1	1.329(7)	C1B	C2B	1.5382
F2	C2	1.328(7)	O1B	C3B	1.5380
F3	C3	1.343(8)	C3B	C4B	1.5381
N2	C7	1.270(7)	C4B	N1B	1.5378
N2	N1B	1.390(10)	N1B	C2B	1.5377
N2	N1A	1.286(9)	C1A	O1A	1.5382
C5	C6	1.401(8)	C1A	C2A	1.5381
C5	C4	1.371(9)	O1A	C3A	1.5377
C6	C1	1.372(8)	C3A	C4A	1.5382
C7	C7[1]	1.434(11)	C4A	N1A	1.5381
C4	C3	1.358(9)	N1A	C2A	1.5376

[1]$-1-x$, $1-y$, $-z$

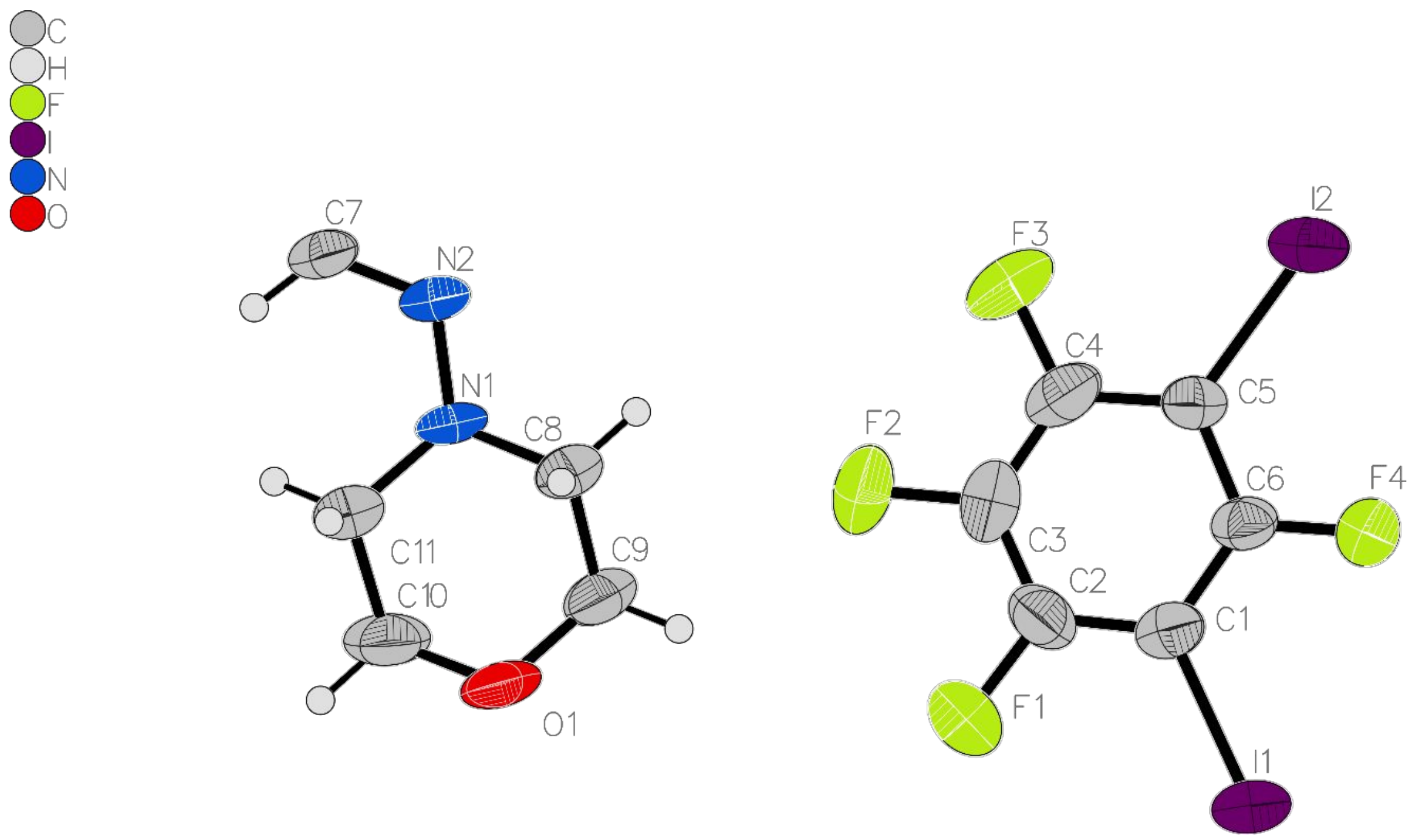

Figure 10 Asymetric unit of **3**·13**tfib**$_2$ showing the atom-labelling scheme. Displacement ellipsoids are drawn at the 50 % probability level, and H atoms are shown as small spheres of arbitrary radius.

Table 13 Bond lengths for **3**·13**tfib**$_2$.

**Atom (A1)**	**Atom (A2)**	***d*(A1 − A2)/Å**	**Atom (A1)**	**Atom (A2)**	***d*(A1 − A2)/Å**
I1	C1	2.086(9)	F2	C3	1.364(12)
I2	C5	2.070(9)	C5	C6	1.408(13)
F4	C6	1.328(13)	C5	C4	1.401(15)
F1	C2	1.357(12)	C1	C6	1.387(14)
F3	C4	1.326(12)	C1	C2	1.369(15)
O1	C9	1.433(14)	C9	C8	1.516(15)
O1	C10	1.423(15)	C11	C10	1.525(14)
N1	N2	1.380(10)	C3	C2	1.368(16)
N1	C11	1.455(14)	C3	C4	1.375(17)
N1	C8	1.451(13)	C7	C7[1]	1.428(17)
N2	C7	1.283(13)			

[1]1−*x*, −*y*, −*z*

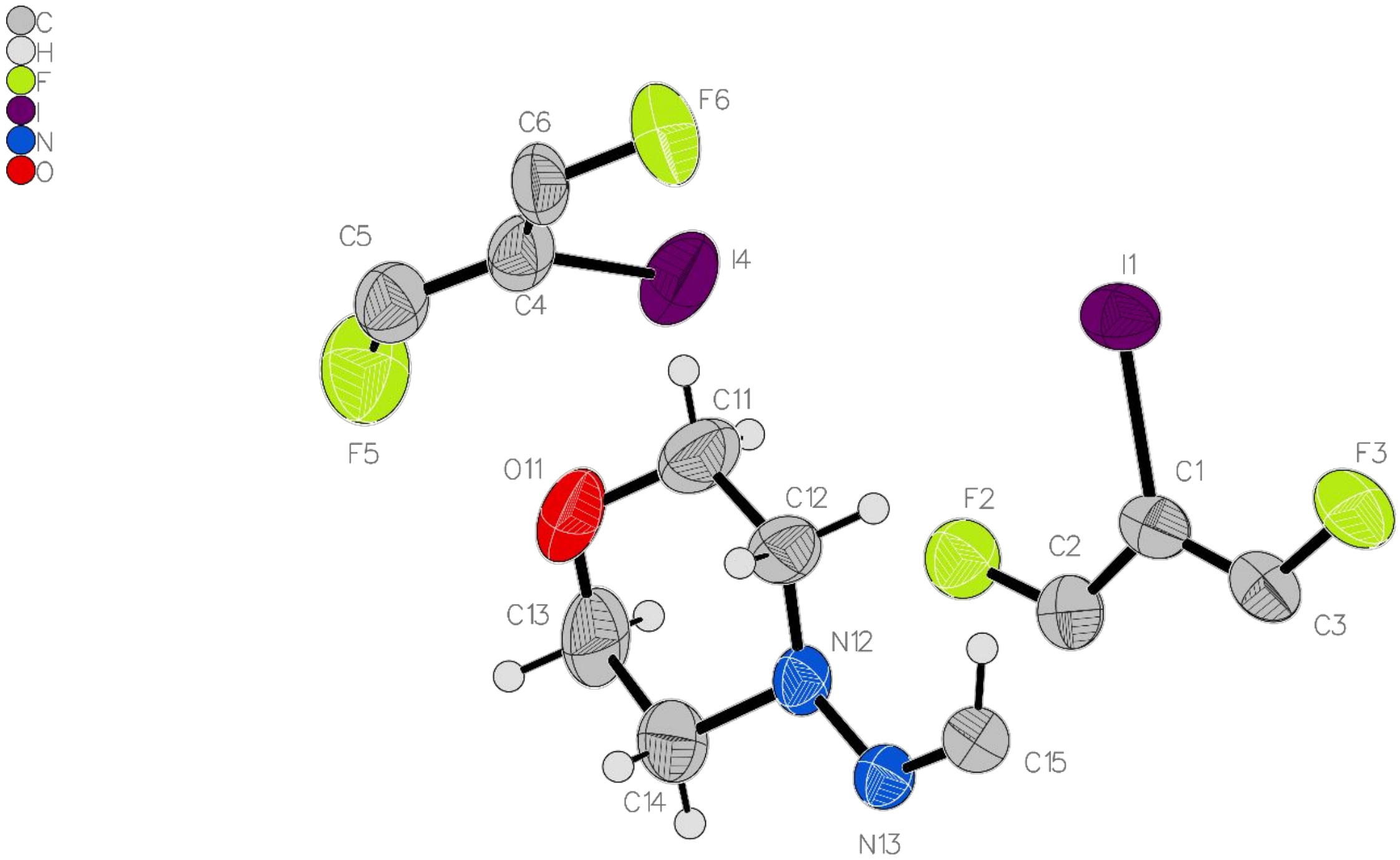

Figure 11 Asymetric unit of **3**·14**tfib**$_2$ showing the atom-labelling scheme. Displacement ellipsoids are drawn at the 50 % probability level, and H atoms are shown as small spheres of arbitrary radius.

Table 14 Bond lengths for **3**·14**tfib**$_2$.

**Atom (A1)**	**Atom (A2)**	***d*(A1 − A2)/Å**	**Atom (A1)**	**Atom (A2)**	***d*(A1 − A2)/Å**
I1	C1	2.079(4)	C15	C15^{1}	1.427(9)
I4	C4	2.079(5)	C1	C2	1.371(6)
F3	C3	1.346(5)	C1	C3	1.380(7)
F2	C2	1.354(6)	C2	C3^{2}	1.368(7)
N13	N12	1.371(5)	C3	C2^{2}	1.368(7)
N13	C15	1.280(6)	C12	C11	1.501(7)
F6	C6	1.349(6)	C4	C5	1.368(7)
N12	C12	1.436(6)	C4	C6	1.369(8)
N12	C14	1.461(6)	C5	C6^{3}	1.356(7)
F5	C5	1.354(6)	C6	C5^{3}	1.356(7)
O11	C13	1.413(7)	C13	C14	1.513(8)
O11	C11	1.419(7)			

^{1}1−*x*, −*y*, 1−*z*; ^{2}2−*x*, −*y*, 1−*z*; ^{3}2−*x*, −1−*y*, 2−*z*

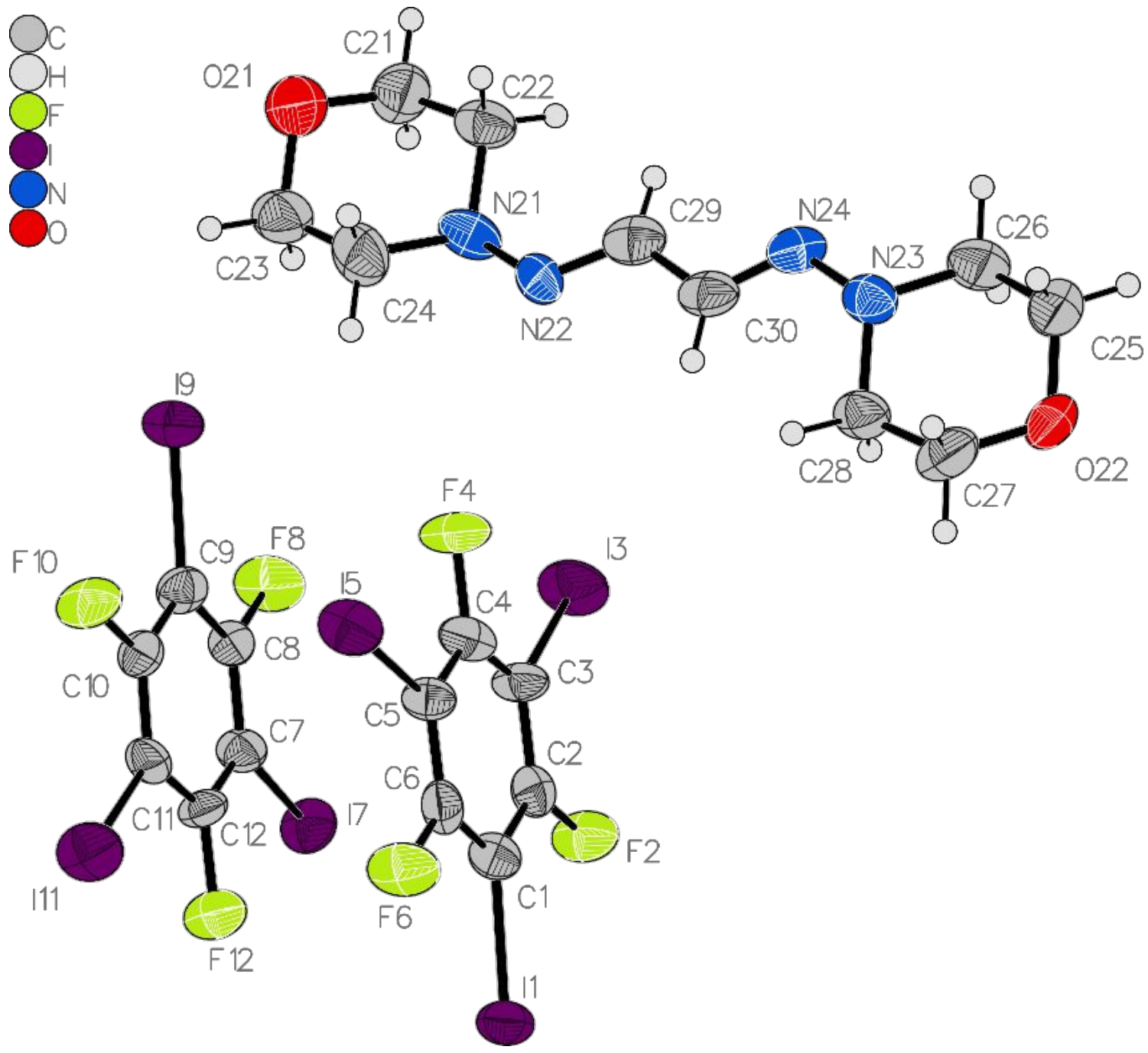

Figure 12 Asymetric unit of **3**·135**tfib**$_2$ showing the atom-labelling scheme. Displacement ellipsoids are drawn at the 50 % probability level, and H atoms are shown as small spheres of arbitrary radius.

Table 15 Bond lenghts for **3**·135**tfib**$_2$.

**Atom (A1)**	**Atom (A2)**	***d*(A1 − A2)/Å**	**Atom (A1)**	**Atom (A2)**	***d*(A1 − A2)/Å**
I1	C1	2.101(10)	N23	C26	1.471(12)
I7	C7	2.090(10)	N21	C22	1.452(12)
I9	C9	2.085(10)	N21	C24	1.472(12)
I5	C5	2.075(10)	C7	C8	1.349(13)
I3	C3	2.075(9)	C7	C12	1.384(12)
I11	C11	2.074(9)	C1	C6	1.358(14)
F2	C2	1.332(11)	C1	C2	1.393(12)
F8	C8	1.354(10)	C8	C9	1.364(13)
F10	C10	1.348(10)	C12	C11	1.392(13)
F12	C12	1.340(10)	C9	C10	1.402(12)
F4	C4	1.336(11)	C5	C6	1.368(13)
F6	C6	1.358(10)	C5	C4	1.383(12)
O22	C25	1.428(13)	C3	C4	1.376(14)
O22	C27	1.414(12)	C3	C2	1.373(13)
N24	N23	1.379(11)	C30	C29	1.417(13)
N24	C30	1.258(11)	C11	C10	1.342(13)
O21	C21	1.423(12)	C22	C21	1.508(14)
O21	C23	1.426(13)	C28	C27	1.502(13)
N22	N21	1.352(10)	C25	C26	1.518(15)
N22	C29	1.294(11)	C23	C24	1.516(15)
N23	C28	1.462(12)			

3. Halogen bond parameter comparisons

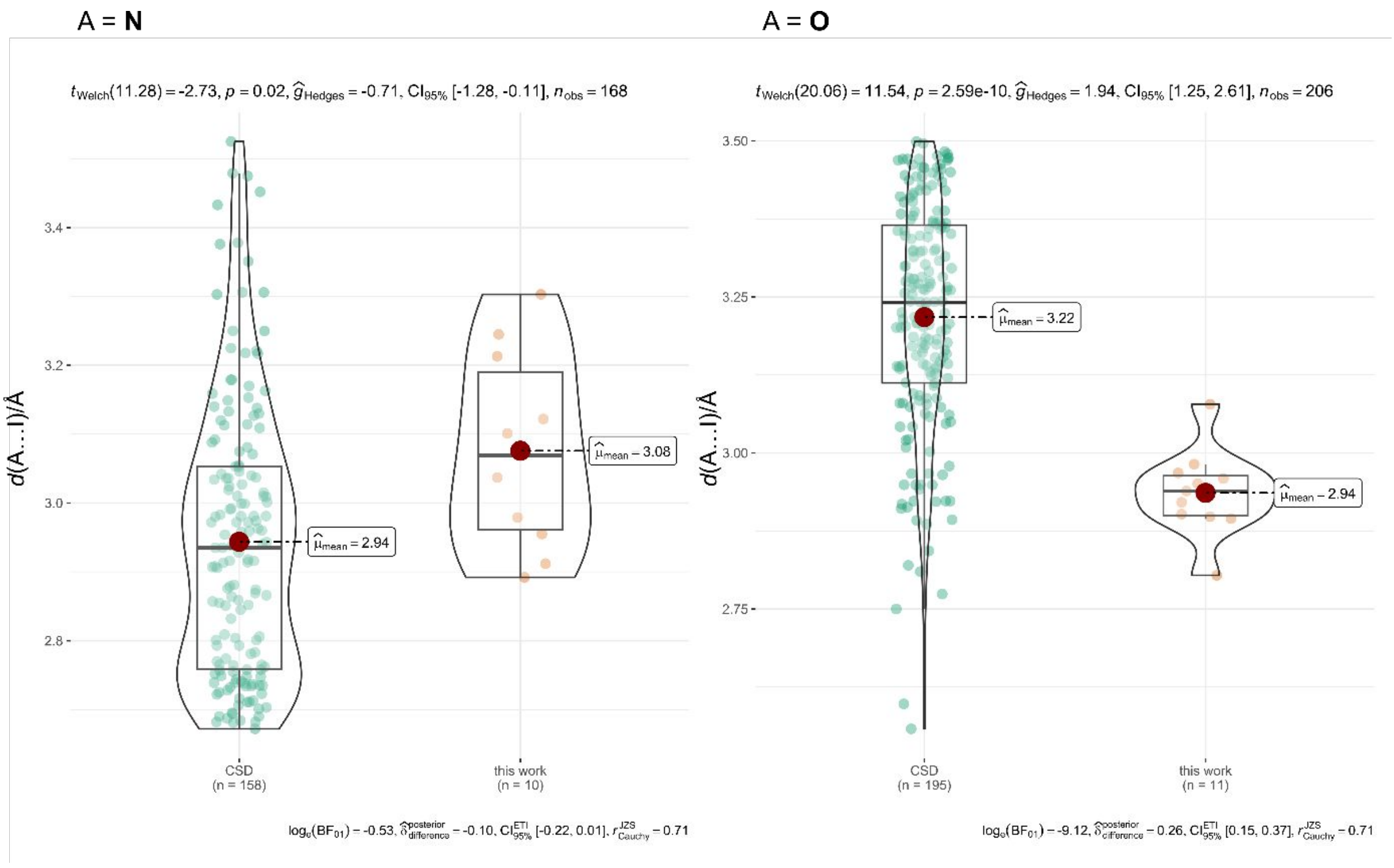

Figure 13 Comparison of the halogen bond lengths (*d*) observed in this work vs. the CSD database.

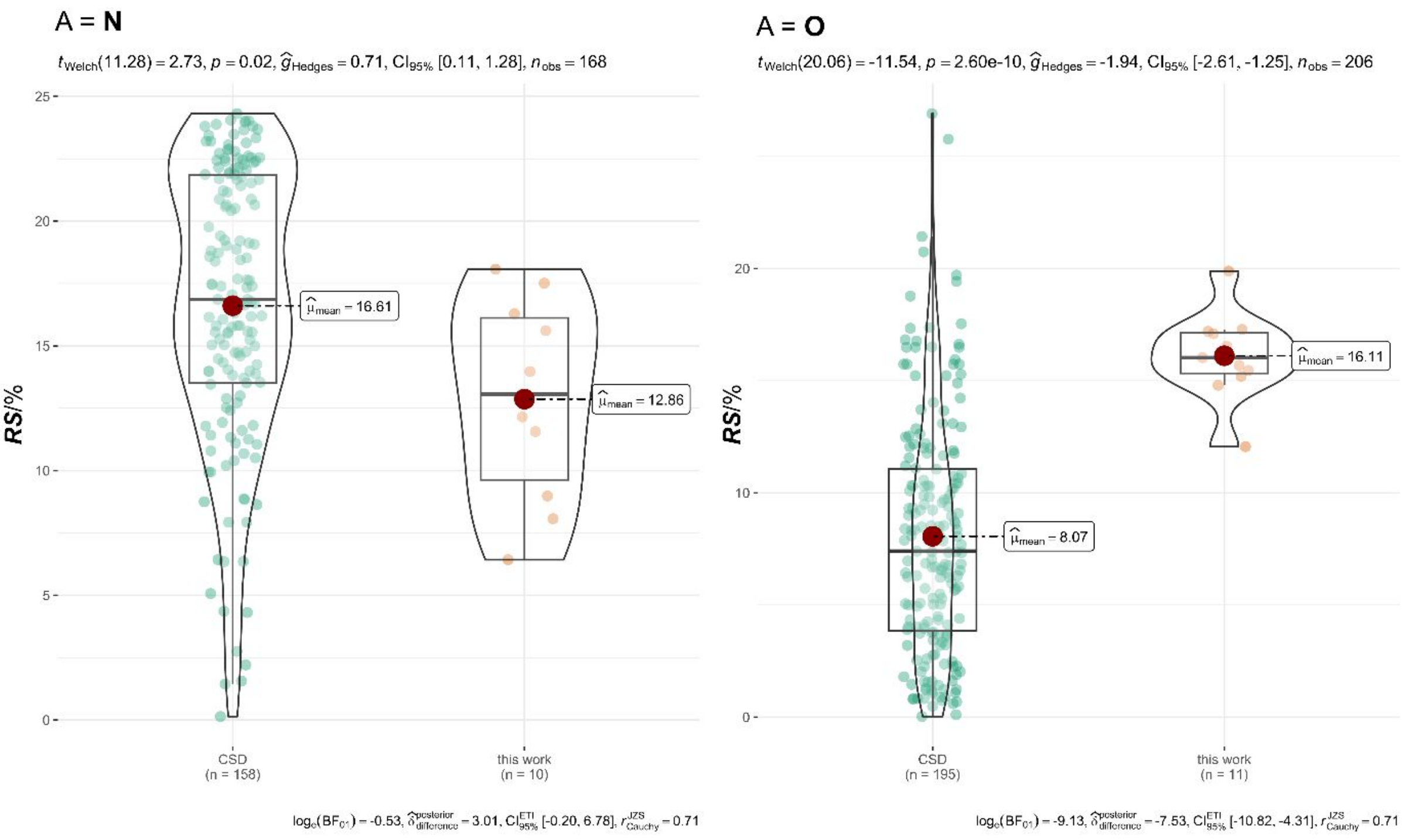

Figure 14 Comparison of the halogen bond relative shortening (*RS*) observed in this work vs. the CSD database.

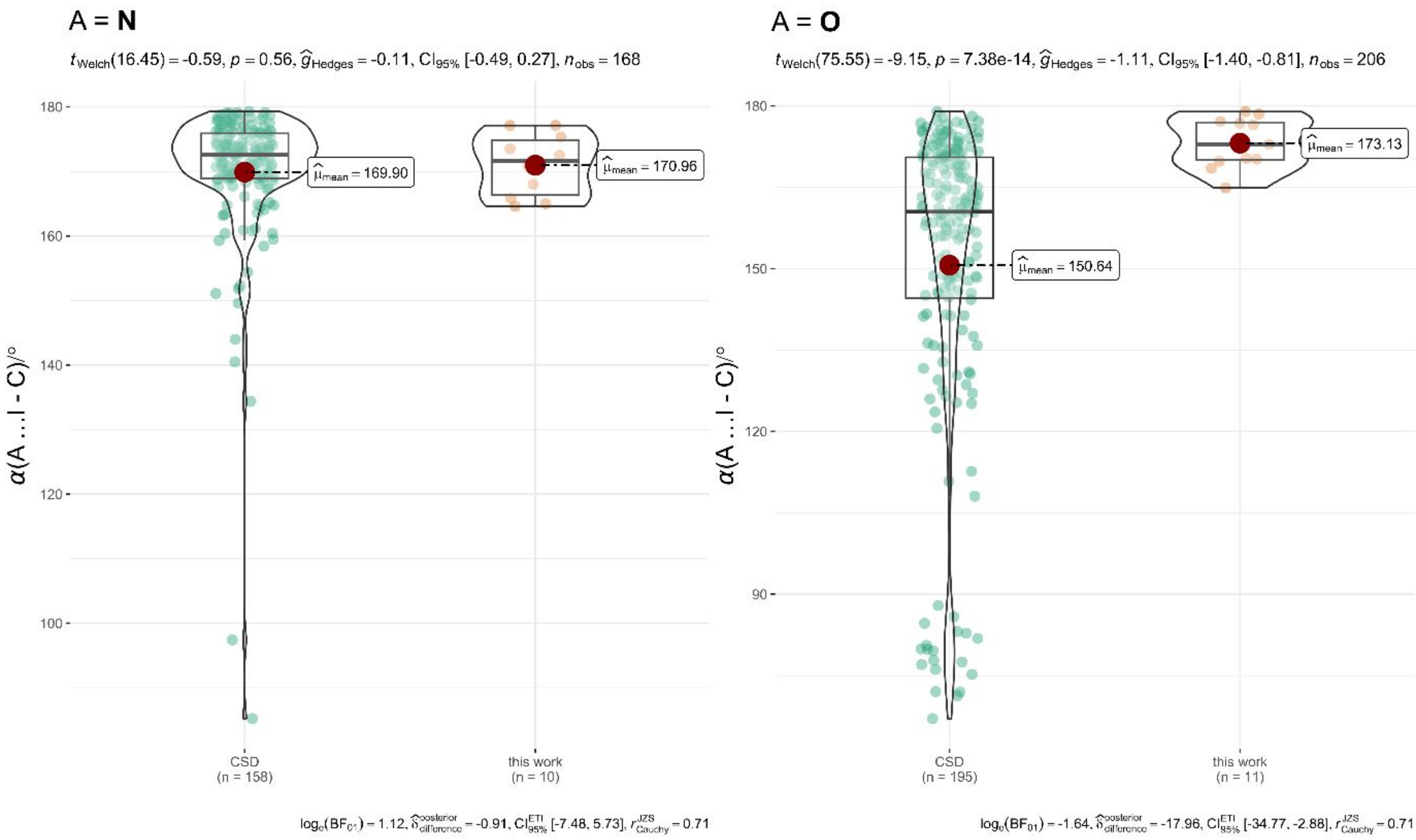

Figure 15 Comparison of the halogen bond angles (α) observed in this work vs. the CSD database.
